# Nucleophiles Target the Tungsten Center Over Acetylene in Biomimetic Models

**DOI:** 10.1021/acs.inorgchem.4c00286

**Published:** 2024-06-14

**Authors:** Miljan Z. Ćorović, Angela Milinkovic, Niklas Stix, Antoine Dupé, Nadia C. Mösch-Zanetti

**Affiliations:** † Institute of Chemistry, Inorganic Chemistry, University of Graz, 8010 Graz, Austria

## Abstract

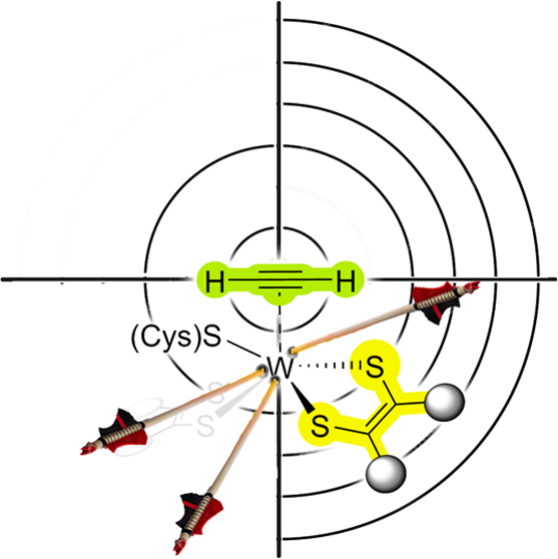

Inspired by the first shell mechanism proposed for the tungstoenzyme acetylene hydratase, the electrophilic reactivity of tungsten-acetylene complexes [W(CO)(C_2_H_2_)(6-MePyS)_2_] (**1**) and [WO(C_2_H_2_)(6-MePyS)_2_] (**2**) was investigated. The biological nucleophile water/hydroxide and *tert*-butyl isocyanide were employed. Our findings consistently show that, regardless of the nucleophile used, both tungsten centers W(II) and W(IV), respectively, are the preferred targets over the coordinated acetylene. Treatment of **2** with aqueous NaOH led to protonation of coordinated acetylene to ethylene, pointing toward the Brønsted basic character of the coordinated alkyne instead of the anticipated electrophilic behavior. In cases involving isocyanides as nucleophiles, the attack on the W(II) center of **1** took place first, whereas the W(IV) complex **2** remained unchanged. These experiments indicate that the direct nucleophilic attack of W-coordinated acetylene by water, as some computational studies of acetylene hydratase propose, is unlikely to occur.

## Abstract 2

Nucleophiles preferentially target the tungsten center instead of coordinated acetylene in acetylene hydratase models. This reactivity causes organometallic transformations of acetylene, such as reduction to ethylene and insertion.

## Introduction

**Figure 1:**
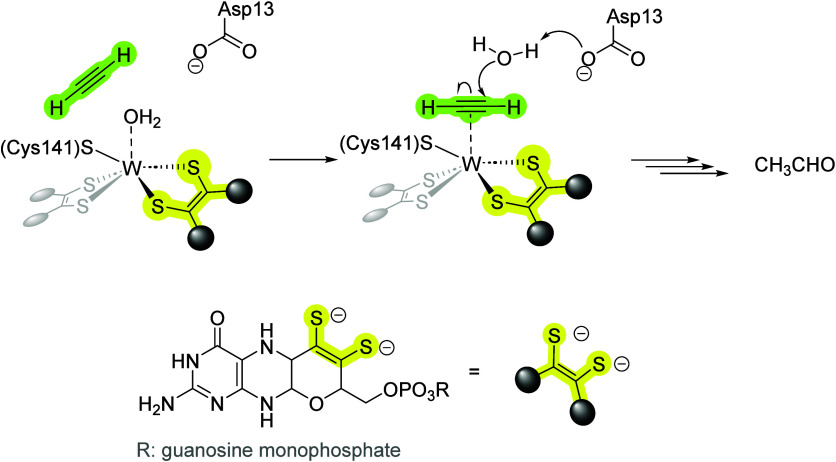
A first step of the first shell AH mechanism, suggested by Himo and co-workers.^3^ Adapted with permission from reference (3). Copyright [2010] Proceedings of the National Academy of Sciences of the United States of America.

Tungsten is a biometal of choice for many bacteria and archaea, mediating crucial redox processes while cycling between oxidation states IV, V, and VI.^1^ Moreover, tungstoenzymes are involved in remarkable organometallic transformations, such as selective hydrogenation of aromatic rings or nonredox acetylene (C_2_H_2_) hydration.^2^ The latter inspired us to study the reactivity of W–C_2_H_2_ adducts, as their formation has been proposed for the initial mechanistic step of acetylene hydratase (AH).^3^ No functional model of this tungstoenzyme has been prepared, probably due to a lack of knowledge about the mechanism. The crystal structure of the enzyme reveals a tungsten(IV) center ligated by two metallopterin cofactors (MPT), one O-ligand (water or hydroxide), and cysteine (Cys141). Near the active site of the enzyme, there is an aspartate (Asp13) crucial for the enzymatic function and a hydrophobic tunnel enabling acetylene to reach the tungsten center.^4^ Numerous contrasting mechanistic possibilities have been suggested for acetylene fermentation in AH, but all can be categorized into two groups.^5^ The first involves the initial coordination of acetylene to W(IV), followed by a nucleophilic attack by water/hydroxide,^3,6,7^ while the second possibility considers coordinated water or hydroxide ions attacking free acetylene.^4,8^ It is challenging to envision a mechanism that does not involve an organometallic intermediate, for two reasons. First, the organometallic chemistry of alkyne adducts of tungsten and its lighter analogue molybdenum is well-established.^9−12^ Second, if the W center exclusively activates the water molecule, other substrates such as nitriles, alkenes, or higher alkynes could potentially undergo hydration, but AH demonstrates selectivity toward acetylene.^13,14^ Therefore, studying bioinspired tungsten complexes is crucial for understanding the role of the metal in the enzymes.^15,16^ Prior to the characterization of the enzyme AH in 2007,^4^ several research groups explored the chemistry of W–C_2_H_2_, offering valuable insights into the reactivity of these compounds. The first tungsten(IV) acetylene adduct, [WO(dtc)_2_(C_2_H_2_)] (dtc = S_2_CNMe_2_, S_2_CNEt_2_), was reported in 1981.^17^ Furthermore, Templeton and co-workers reported a W(IV) compound with scorpionate-based ligands, incorporating AH substrates, acetylene, and water. Notably, no acetaldehyde formation was detected in their experiments.^18^ Therefore, inspired by the first shell mechanism suggested by Himo and co-workers in 2010 (Figure 1),^3^ reactivity patterns of W–C_2_H_2_ complexes were investigated in our group.

**Scheme 1:**
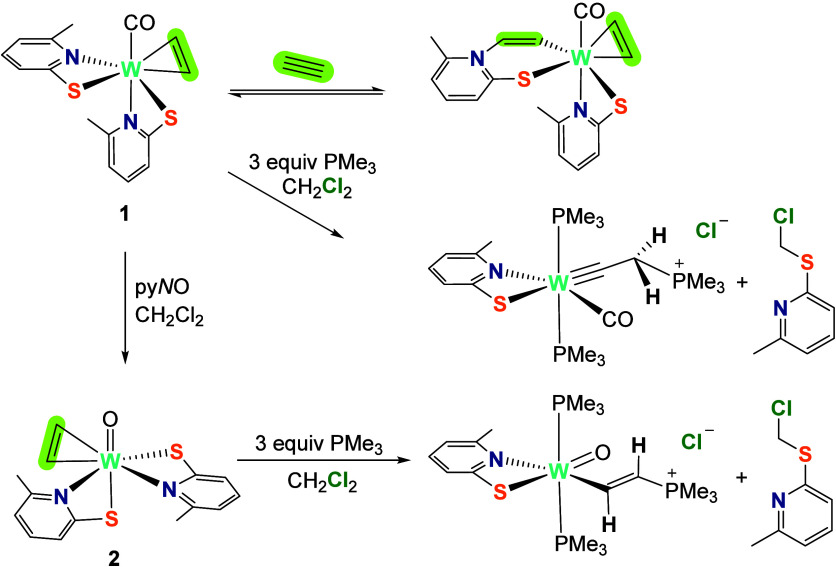
Reaction Scheme Showing the Reactivity of Tungsten Acetylene Adducts **1** and **2**;^19^ Adapted from Reference (19), Copyright [2021] American Chemical Society

Previously, we introduced a fully characterized W(IV) system capable of reversible acetylene binding.^11^ Subsequent work led to the development of synthetic routes to a series of W–alkyne adducts and exploration of their reactivity toward nucleophiles. Our attempts to react the alkyne adducts with water failed, and further reactivity studies of model complexes are required to reach the bioinspired reactivity with water. Interestingly, complex [W(CO)(C_2_H_2_)(6-MePyS)_2_] (**1**) (6-MePyS = 6-methylpyridine-2-thiolate) was found to react with excess of acetylene (Scheme 1),^19^ leading to an insertion of an additional acetylene molecule into the W–N bond of the ancillary ligand—a reactivity considered as nucleophilic attack. Furthermore, we succeeded in reacting alkyne complex **1** and its oxido variant [WO(C_2_H_2_)(6-MePyS)_2_] (**2**) with an excess of PMe_3_ to yield carbyne and η^1^-vinyl complexes, respectively (Scheme 1).^19^

In the reactions with PMe_3_, initial attack most likely occurs at the W center, as such behavior was shown with complexes bearing higher alkynes.^20^ Similarly, hydride addition to cationic Mo diphenylacetylene adduct [CpMo(OPMe_3_)_2_(PhC_2_Ph)]^+^ led to an η^2^-vinyl complex via initial Mo–H bond formation.^21^ To overcome the initial nucleophile coordination, Davidson et al. employed ^*t*^BuNC (isocyanide) and obtained an η^2^-vinyl complex starting from W(II) adducts of hexafluoro-2-butyne.^22^ To our knowledge, no literature data on isocyanide interactions with nonsubstituted acetylene were reported. An alternative approach to enhance the attack on the acetylene carbon could be the indirect activation of the W=O moiety. It is well-known that Lewis acids can interact with metal oxido moieties via binding to the oxido ligand and increasing the electrophilicity of the metal center.^23,24^ Interestingly, the redox activity of the biomimetic Mo(IV) oxido complex was enhanced after adding Sc^3+^ ions, which allowed stoichiometric nitrate reduction.^25^ In our group, particular interest was dedicated to the interaction of metal oxido complexes and highly electrophilic and sterically encumbered B(C_6_F_5_)_3_.^26^ Nevertheless, there is a lack of available data regarding the behavior of π-bound ligands upon activation of the neighboring metal oxido bond.

Herein, we employ three distinct approaches to attempt the desired acetylene activation in biomimetic tungsten complexes [W(CO)(C_2_H_2_)(6-MePyS)_2_] (**1**) and [WO(C_2_H_2_)(6-MePyS)_2_] (**2**):^19^ (a) reactions with water/hydroxide, (b) addition of ^*t*^BuNC as a C-nucleophile, and (c) addition of the B(C_6_F_5_)_3_ as a Lewis acid to activate W(IV) oxido bond. Contrasting the naturally occurring dithiolene-based ligands (Figure 1), the 6-methylpyridine-2-thiolate ligands used are redox innocent. Nonetheless, 6-methylpyridine-2-thiolate ligands may tautomerize to the 6-methylpyridyl-2-thione form, which influences their hardness and, thus, their capability to stabilize different oxidation states. Although apparently different, they are sulfur-rich, may adjust to various coordinative environments, and readily support acetylene coordination at tungsten, allowing further reactivity studies.

## Results and Discussion

### Reaction of W(IV) Alkyne Complexes with Water/Hydroxide Leading to Olefin Formation

**Scheme 2:**
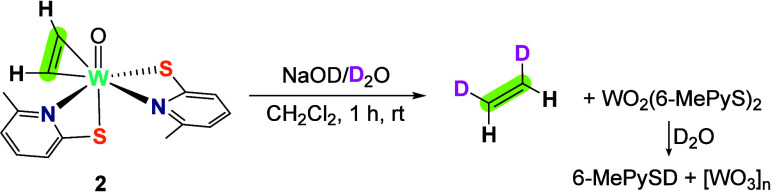
Reduction of Acetylene to Ethylene by Reaction of the Tungsten Complex **2** under Aqueous Basic Conditions at RT

Complexes [W(CO)(C_2_H_2_)(6-MePyS)_2_] (**1**) and [WO(C_2_H_2_)(6-MePyS)_2_] (**2**), respectively, were dissolved in CH_2_Cl_2_, mixed with 1 M aqueous NaOH, and vigorously stirred for 1 h. IR spectroscopic analysis of the atmosphere above the reaction mixture containing complex **2** showed a stretching at 950 cm^–1^ assignable to H–C–H out-of-plane wagging specific for ethylene (literature value: 949 cm^–1^).^27^ To confirm the olefin formation, solutions of **1** and **2**, respectively, in CD_2_Cl_2_, were mixed with a solution of NaOD in D_2_O in J. Young tubes at rt, and ^1^H NMR spectra were recorded after 1 h. While complex **1** remained stable under these conditions, the ^1^H NMR spectrum (Figure S2) of the reaction mixture containing complex **2** revealed partial conversion of the starting compound to *cis*-*d*_2_-ethylene^28^ C_2_H_2_D_2_ (5.40 ppm), as shown in Scheme 2. The formation of ethylene is accompanied by partial decomposition of the starting compound, as a signal at 2.41 ppm could be assigned to the methyl group of the protonated ligand 6-MePySD (Figure S2).

In contrast, adding water to CD_2_Cl_2_ solutions of complexes **1** and **2**, respectively, led only to slow protonation of the bidentate 6-MePyS ligands, as observed via ^1^H NMR spectroscopy, demonstrating that nucleophilic hydroxide is required for ethylene formation.

**Scheme 3:**
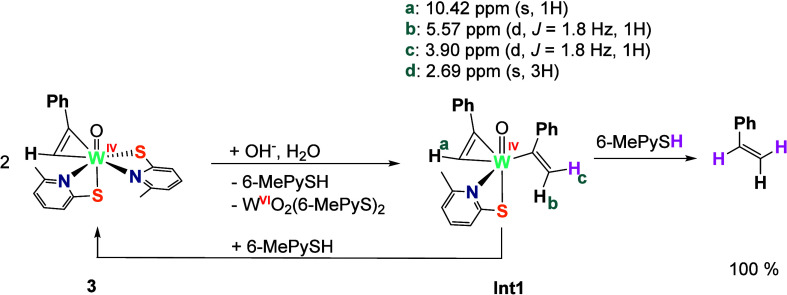
Reaction of the Complex **3** with Aqueous Base, Leading to the Quantitative Formation of Styrene

Since no intermediates were detected in the reaction of **2** with an aqueous base, we explored a comparable reaction with a tungsten(IV) complex bearing the substituted alkyne, phenylacetylene (PhC_2_H). Thus, an oxygen-free 15% NaOH/water solution was added to a CH_2_Cl_2_ solution of [WO(HC_2_Ph)(6-MePyS)_2_]^20^ (**3**) and vigorously stirred for 1 h at rt. The yellow color of the CH_2_Cl_2_ layer faded over the reaction time. GC-MS analysis of the organic layer showed quantitative conversion of the coordinated phenylacetylene to (free) styrene (compared to mesitylene used as the internal standard). Degassing the NaOH solution prior to reaction is essential, as adding the benchtop NaOH solution leads to the formation of phenylacetylene as the only organic product. Interestingly, upon addition of O_2_ to CD_2_Cl_2_ or CDCl_3_ solutions of complex **3**, no reactivity was observed, implying a significant role of the hydroxide. For this reason, stoichiometric studies with NaOH were carried out to take a closer look at the formation of styrene derived from phenylacetylene complex **3**. Thus, a CDCl_3_ solution of complex **3** and an aqueous NaOH solution (2.0 equiv) were mixed in a J. Young tube, and the reaction was followed by ^1^H NMR spectroscopy. After 1 h, the ^1^H NMR spectrum (Figure S3, Table S2) reveals the presence of styrene, free ligand 6-MePySH, and the intermediate tungsten(IV) complex [WO(PhC_2_H)(C(Ph)=CH_2_)(6-MePyS)] (Int**1**), alongside the unreacted starting complex **3**. It is worth remarking that complex **3** exhibits two isomers with respect to the phenylacetylene and gives rise to two singlets in the downfield region. Surprisingly, Int**1** shows resonances belonging to a tungsten complex bearing two unsaturated moieties derived from phenylacetylene. A singlet flanked with ^183^W satellites at 10.42 ppm belongs to the acetylenic proton of the coordinated phenylacetylene, while two doublets resonating at 5.57 and 3.90 ppm (*J*_H–H_ = 1.8 Hz) correspond to the geminal vinylic protons of the 1-phenylvinyl ligand.^29,30^ The two doublets exhibit a crosspeak in the ^1^H–^1^H COSY spectrum, as well as two crosspeaks with the same carbon atom (107.73 ppm) in the ^1^H–^13^C HSQC spectrum (see Figure S6). Unlike the starting complex **3**, Int**1** bears only one bidentate 6-MePyS. Labeling experiments using a NaOD/D_2_O solution revealed the absence of the peak at 3.90 ppm (red proton H_c_ in Int**1**, Scheme 3), alongside the multiplicity reduction of the signal H_b_ resonating at 5.57 ppm from a doublet to a singlet (see Figure S4, Table S3). Furthermore, *trans*-α,β-*d*_2_-styrene is formed as a major product, together with a small amount of the *cis*-isomer of the α,β-*d*_2_-styrene.^31^ Measurements of the same reaction solution after 30 h revealed the disappearance of the Int**1** signals and the increase of styrene signals, as shown in Figure S5. Complete conversion to styrene was not observed in any NMR experiment, presumably due to the absence of phase mixing in the NMR tube. Due to the presence of two unsaturated moieties deriving from phenylacetylene in Int**1**, it is assumed that the initial attack of complex **3** by hydroxide causes formation of a binuclear tungsten complex with a bridging vinyl group μ-CH=CH(Ph). Depending on the orientation, a *cis* or *trans* isomer can be formed consistent with the observed two *d*_2_-isomers of styrene. Such a dimeric species would undergo swift decomposition to Int**1** and the corresponding dioxido complex [WO_2_(6-MePyS)_2_], the latter being the driving force of the overall process due to the thermodynamically favorable W=O bond formation.^32^ The formation of [WO_2_(6-MePyS)_2_] has been indirectly confirmed via its decomposition products in the presence of water, which are protonated ligand 6-MePySH and undefined [WO_3_]_*n*_ species.^33^ An independent NMR study confirmed the formation of [WO_3_]_*n*_ species from the dioxido complex under aqueous basic conditions (see SI). Quantitative conversion of the starting complex to styrene indicates that the vinyl intermediate Int**1** must react with 6-MePySH to recover one equivalent of complex **3**.

During the reaction with the aqueous base, the tungsten(IV) center undergoes oxidation and provides the two electrons necessary for the phenylacetylene reduction. As shown in Scheme 3, only 0.5 equiv of the NaOH is necessary for the formation of Int**1**, yet following the reaction under substoichiometric conditions was extremely slow, and attempts to isolate pure Int**1** failed. Complex **3** did not react with Bu_4_NOH in organic solvents as demonstrated by NMR spectroscopy, most likely due to the absence of a proton necessary for Int**1** formation. The carbonyl phenylacetylene complex [W(CO)(HC_2_Ph)(6-MePyS)_2_]^20^ did not react with basic aqueous solutions to styrene, a similar behavior as was observed with complex **1** described above. The formation of styrene only when using a degassed NaOH solution indicates the reactivity of compound **3** with dioxygen but only in water, while no reactivity has been noticed in dry NMR solvents.

As demonstrated above, the tungsten centers in complexes **2** and **3** act as electrophiles, a reactivity observed previously with phosphines^19,20^ and described below with isocyanides. Schrock and co-workers found similar reactivity for the tungsten(IV) diphenylacetylene complex [W(C_2_Ph_2_)(pin)_2_] (pin = pinacol dianion), which reacted with pinacol to [W(pin)_3_] and *cis*-stilbene.^34^ Moreover, similar to our d^2^ W(IV) complexes, the isoelectronic metals Nb(III) and Ta(III) can form alkyne adducts that give olefins upon quenching with water.^35,36^ The formation of vinyl intermediates has also been proposed in the case of Nb(III) and Ta(III), but lacking isolation. Although envisioned as targets for nucleophilic attack of hydroxide/water, tungsten(IV) coordinated alkynes act as vicinal olefin dianion synthons and could be used as an alternative to dilithioolefins.^37,38^ Such a binding mode allows for stoichiometric hydrogenation of alkynes in water under mild conditions. This is consistent with a high contribution of the metallacyclopropene resonance structure according to the Dewar–Chatt–Duncason alkyne bonding model.^39^ The observed absence of hydrogenation of the coordinated alkyne in water may be explained as follows: Only under basic conditions the intermediately formed hydroxido species is deprotonated under the formation of W–oxido bonds, which drives the overall reaction. Finally, to reduce the alkyne to an olefin, the former must be coordinated to a d^2^ metal center, as indicated by our results and by examples from literature.^34−36^ As the overall reaction of AH is redox-neutral, the herein reported redox reaction of acetylene adducts with an aqueous base does not mimic biological behavior.

### Reactions of W(II) Acetylene Complex with Isocyanide Lead to Acetylene Insertion

**Scheme 4:**
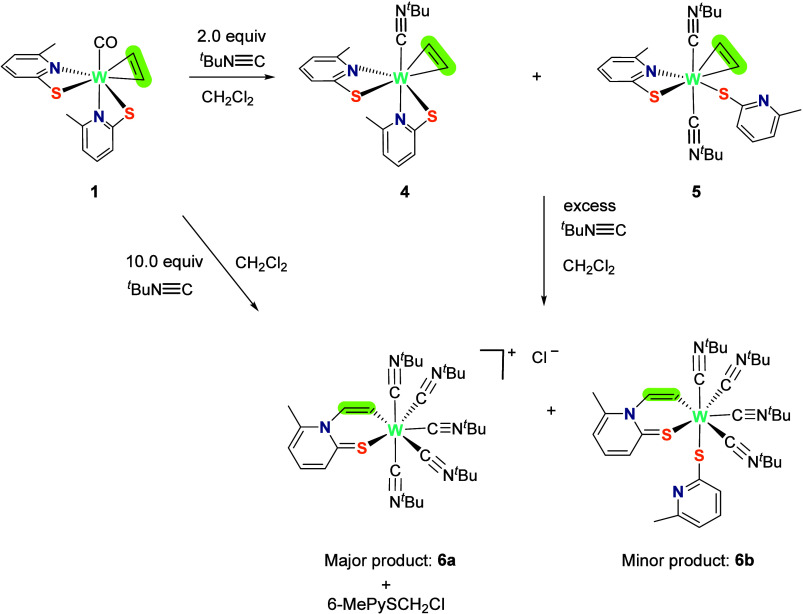
Reaction of **1** with *tert*-Butyl Isocyanide Leads to the Formation of Acetylene Compounds **4** and **5**; Further Addition of the Isocyanide Promotes Acetylene Insertion into the W–N Bond and Presumably the Formation of Inserted Complexes **6a** and **6b**

To attempt the direct nucleophilic attack on the coordinated acetylene, complexes **1** and **2** were reacted with isocyanide as a potent nucleophile. No reactivity was observed with complex **2** under various conditions. On the other hand, the dropwise addition of a *tert*-butyl isocyanide solution (2 equiv) to a CH_2_Cl_2_ solution of **1** and subsequent stirring for 6 h leads to a color change from violet to brown. After the workup, two tungsten species could be isolated and identified. The brown solids were characterized as [W(CN^*t*^Bu)(C_2_H_2_)(6-MePyS)_2_] (**4**) and [W(CN^*t*^Bu)_2_(C_2_H_2_)(6-MePyS)(*S*–6-MePyS)] (**5**), as depicted in Scheme 4.

From the obtained mixture of **4** and **5**, the former could be isolated in 18% yield as a light brown powder and **5** in 48% yield as a brown powder as described in the SI. Selective formation was not possible, as ^1^H NMR spectroscopy measurements of reaction mixtures containing **1** and varying equivalents of *tert*-butyl isocyanide (ranging from 0.5 to 3.5 equiv) demonstrated the consistent formation of mixtures comprising compounds **4** and **5**. NMR, IR spectroscopy, and mass spectrometry confirmed a monoisocyanide complex **4** with both 6-MePyS ligands coordinated in a bidentate fashion via the S and N atom to the metal center and only one isocyanide ligand as shown in Scheme 4. In the ^1^H NMR spectrum of **4**, the acetylenic protons appear as a broad singlet at 13.40 ppm in CD_2_Cl_2_ at room temperature, while all other signals are sharp. The two methyl groups of the two 6-MePyS ligands are detected at 1.90 and 1.14 ppm, whereas the ^*t*^Bu group resonates at 1.45 ppm. When the CD_2_Cl_2_ reaction mixture is cooled to −40 °C, the singlet for the acetylene ligand is split into two singlets (Figure 3: 13.87 and 12.98 ppm). This points toward alkyne rotation in complex **4** at rt, contrasting the more rigid coordination at the corresponding carbonyl complex **1**. Line shape analysis indicates that at rt the free energy of rotation of the acetylene ligand in **4** is 13.4 kcal/mol (see SI), which falls in the range of reported tungsten carbonyl acetylene complexes in literature.^10^ This dynamic behavior is supported by the ^13^C NMR spectrum, where the acetylene carbons cannot be detected with the achieved signal-to-noise ratio. This also refers to the quarternary carbon in M–C≡N, which is obscured due to the long relaxation time.^40^ All other carbon atoms for two 6-MePyS ligands and the ^*t*^Bu group (63.09 and 31.32 ppm) are detected. The isocyanide ligand can be further observed via IR spectroscopy, where two bands at 2020 cm^–1^ (w) and 1908 cm^–1^ (s) are indicative of the M–C≡N moiety^41^ with a CN triple bond character. The M–C=N double bond is usually detected at ν = 1500–1600 cm^–1^,^42^ and compared to free ^*t*^BuNC (ν_CN_ = 2137 cm^–1^)^43^ the bands are shifted toward lower wavenumber.

**Figure 2:**
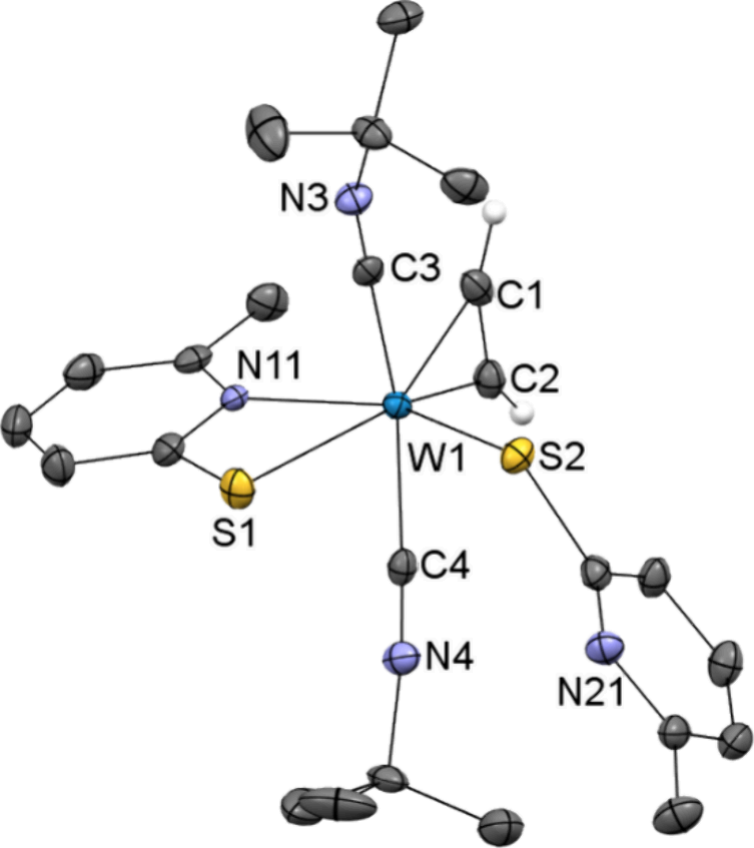
Molecular structure of [W(^*t*^BuNC)_2_(C_2_H_2_)(6-MePyS)(*S*-6-MePyS)] (**5**) showing the atomic numbering scheme. The ellipsoids are drawn at the 50% probability level. Except for those of the acetylene group, H atoms were omitted for clarity.

We were able to obtain single crystals suitable for X-ray diffraction analysis of **5**, revealing a heptacoordinate tungsten(II) center with one of the pyridine ligands only coordinated via the thiolato group [W1–S2 2.3828(10) Å, C22–S2–W1 112.46(12)°] (Figure 2). The two S atoms are *cis* to each other with an angle close to 90° [S1–W1–S2 88.95(3)°], and two *tert*-butyl isocyanide ligands are bonded to the metal *trans* to each other [C3–W1–C4 161.15(14)°], eclipsed to the acetylene ligand. The distance between the tungsten center and the acetylenic C1–C2 bond in complex **5** [1.92355(8) Å] is slightly shorter compared to the starting CO-complex [1.930(4) Å]^19^ or the W–CN^*t*^Bu bond [W–C3 2.083(4) Å, W–C4 2.111(4) Å]. The short distances of 1.290(5) Å between C1–C2 as well as 1.165(5) and 1.159(4) Å for C3–N3 and C4–N4 confirm their multiple bond character, respectively. In the tris-isocyanide cation Mo(CN^*t*^Bu)_3_(^*t*^BuHNCCNH^*t*^Bu)(bpy)]^2+^, similar values are reported, such as the bond lengths of Mo–C1 [2.03(1) Å], Mo–C3 [2.10(1) Å], C1–C1′ [1.38(1) Å], and C3–N3 [1.15(2) Å].^44^

**Figure 3:**
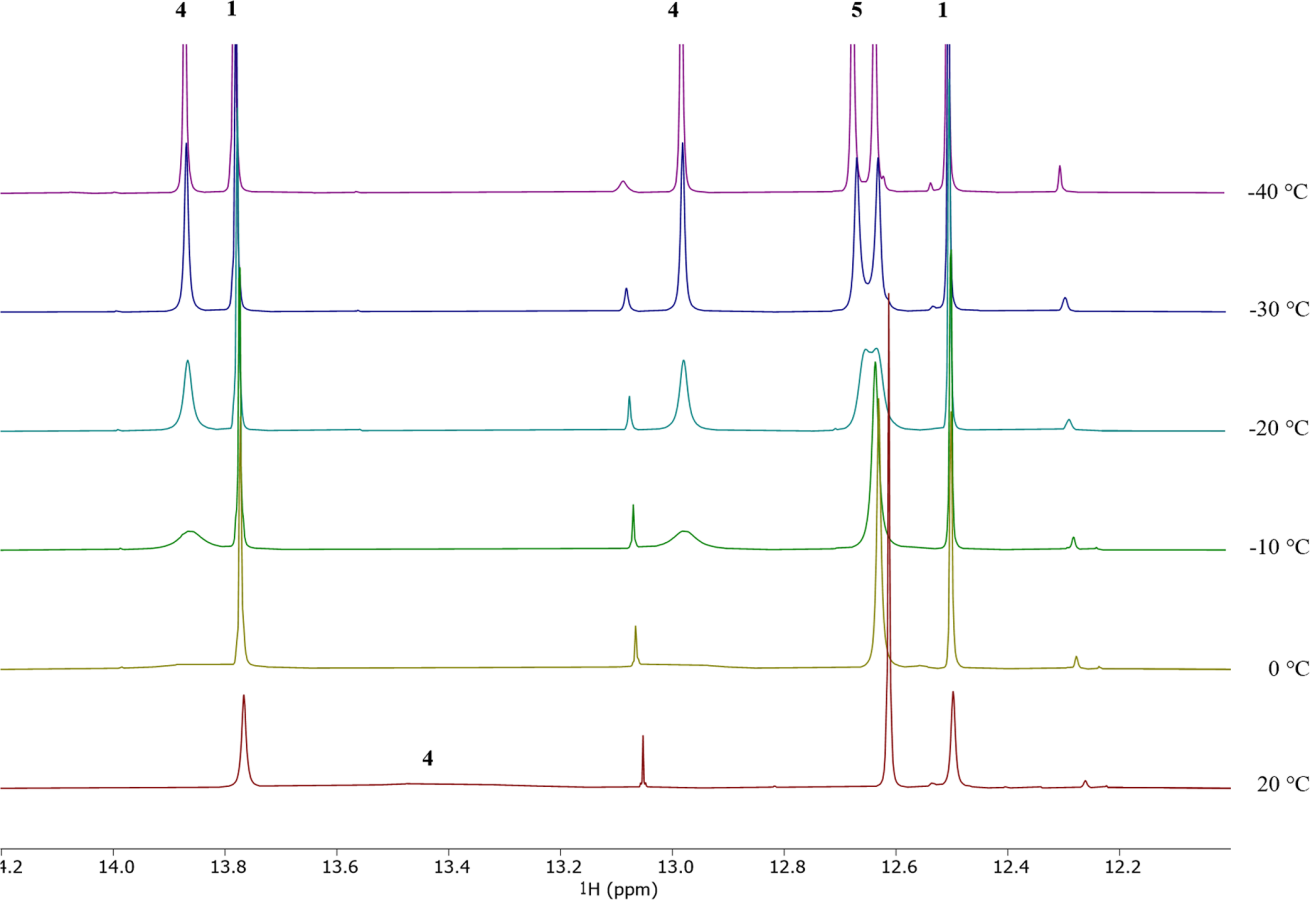
VT-NMR spectra showing the area of acetylene protons of [W(CO)(C_2_H_2_)(6-MePyS)_2_] (**1**), [W(CN^*t*^Bu)(C_2_H_2_)(6-MePyS)_2_] (**4**), and [W(CN^*t*^Bu)_2_(C_2_H_2_)(6-MePyS)(*S*–6-MePyS)] (**5**). The integral ratio between species **4** and **5** does not change upon variation of the temperature. For full VT-NMR spectra, see SI, Figure S11. Reaction conditions: 1 equiv of [W(CO)(C_2_H_2_)(6-MePyS)_2_] (20 mg, 0.04 mmol) and 2 equiv of *tert*-butyl isocyanide (9 μL, 0.08 mmol) in CD_2_Cl_2_ were kept at rt for 18 h. The unmarked small peaks at 13.05 and 12.26 ppm belong to a product of a subsequent reaction (vide infra).

The ^1^H NMR spectrum of **5** in CD_2_Cl_2_ recorded at rt displays very broad peaks for the 6-MePyS ligands (7.65–6.21 ppm for aromatic protons, 2.39 ppm for CH_3_) pointing toward a dynamic behavior of the two 6-MePyS ligands. This is consistent with the solid-state structure where one pyridine thiolate ligand is coordinated only via the sulfur atom. The acetylene protons (12.61 ppm) and ^*t*^Bu groups (1.37 ppm) give sharp signals. The characteristic two singlets for the HC≡CH ligand (in the carbonyl complex **1** at 13.77 and 12.50 ppm) are not observed in the bis-isocyanide complex **5**. Upon cooling the sample to −40 °C, the acetylenic protons’ signal (12.61 ppm) is split into two singlets (Figure 3: 12.68 and 12.64 ppm). The ligands’ dynamic behavior is evident in the ^13^C NMR spectrum at rt (Figure S10). Only specific carbon atoms, such as the acetylene carbons at 195.81 ppm, the quaternary carbon at 57.53 ppm in the ^*t*^Bu group of the coordinated isocyanides, and the six identical methyl groups at 31.31 ppm of the two ^*t*^Bu groups, show detectable signals, while others are obscured. According to IR spectroscopy, the bands for the W–C≡N moiety are slightly shifted to higher frequency compared to the mono species, namely to 2128 and 2036 cm^–1^, aligning with literature data.^45^

If a CH_2_Cl_2_ solution of **1** is treated with an excess of isocyanide and followed by ^1^H NMR spectroscopy, it is indicative that with increasing amounts of *tert*-butyl isocyanide (>3 equiv), immediate further conversion of **4** and **5** to two new species **6a**+**6b** in the ratio 4:1 occurred (see Scheme 4). Moreover, ^1^H NMR spectroscopy of the isolated mixture of **6a** and **6b** points toward the formation of two tungsten compounds in which insertion of acetylene into the W–N bond of the 6-MePyS ligand occurred, namely ([W(*C*,*S*–CHCH–*N*–6-MePyS)(CN^*t*^Bu)_5_][Cl], **6a**, major product) and [W(*C*,*S*–CHCH–*N*–6-MePyS)(*S*–6-MePyS)(CN^*t*^Bu)_4_], **6b**, minor product) as shown in Scheme 4. Next to the inserted C,S ligand, both complexes **6a**+**6b** are coordinated by several molecules of isocyanide, while they differ in the second pyridine thiolate ligand. In the major product **6a**, the latter is displaced by an additional molecule of isocyanide upon reaction with the solvent CH_2_Cl_2_, forming 6-MePySCH_2_Cl, while in the minor product (**6b**), the second thiolate ligand is still coordinated. The ancillary ligand is known to react with CH_2_Cl_2_ as previously described (see Scheme 1).^19^

The inserted acetylene in **6a** is evidenced by its ^1^H NMR spectrum, which exhibits two doublets at 8.53 ppm (*J*_H–H_ = 15.3 Hz) and 7.15 ppm (*J*_H–H_ = 15.3 Hz) with a ratio of 1:1 as well as by ^1^H–^1^H COSY and ^1^H–^13^C HSQC spectra. The data correspond to the literature-known iron complexes bearing inserted acetylene.^46^ Further ^1^H NMR data indicates the presence of only one 6-MePyS moiety in **6a** (see SI, Figure S12), while the exact number of coordinated isocyanide ligands cannot be deduced from the spectral data only. Also, for **6b**, in the ^1^H NMR spectrum, two doublets at 7.93 ppm (*J*_H–H_ = 10.6 Hz) and 6.61 ppm (*J*_H–H_ = 10.6 ppm) in a 1:1 ratio are assigned to the inserted acetylene ligand. We have previously observed the insertion of acetylene into the W–N bond, forming complexes of the type [W(CO)(C_2_H_2_)(HCCH-6-RPyS)(6-RPyS)] (R = H, Me), and the inserted C_2_H_2_ molecule exhibits similar spectroscopic data (see Scheme 1).^19,47^ Therefore, minor product **6b** is most likely a neutral tetrakis(*tert*-butyl isocyanide)W(II) complex with an inserted acetylene ligand into the W–N bond of one 6-MePyS ligand. The difference in coupling constant (**6a**: *J*_H–H_ = 15.3 Hz vs **6b**: *J*_H–H_ = 10.6 Hz) could be attributed to the difference in the charge of the complexes. Specifically, major product **6a** features a tungsten(II) cation, potentially contributing to the observed increase in the coupling constant. The deconvoluted ^1^H NMR spectra of the mixture containing **6a** and **6b** are presented in Figure S14 (SI). IR spectrum of **6a**+**6b** displays sharp bands for M–C≡N oscillations at 2091 and 2033 cm^–1^ as well as an additional signal at 1847 cm^–1^, similar to IR values for W(^*t*^BuNC)_6_ (1960 cm^–1^, 1856 cm^–1^).^42^

**Figure 4:**
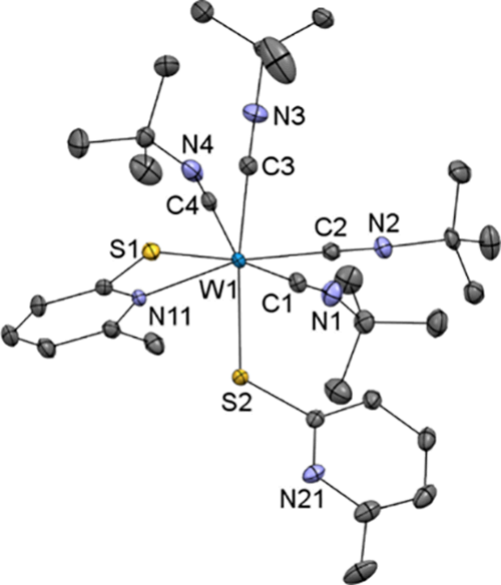
Molecular structure of [W(^*t*^BuNC)_4_(6-MePyS)(*S*–6-MePyS)] (**6c**). The ellipsoids are drawn at the 50% probability level. The H atoms were omitted for clarity.

On a single occasion, single crystals suitable for X-ray diffraction analysis were obtained from a reaction of **1** with an excess of isocyanides. The analysis revealed the formation of [W(CN^*t*^Bu)_4_(6-MePyS)(*S*–6-MePyS)] (**6c**, Figure 4), a heptacoordinated W(II) compound bearing four isocyanide ligands and no acetylene. Moreover, only one 6-MePyS ligand is bound in the bidentate fashion, and the other only via the S atom of the ligand. The red crystals were analyzed via ^1^H NMR and ^13^C NMR spectroscopy in CD_3_CN, revealing only one set of protons belonging to the bidentate ligand, contrasting the XRD results. The obtained data suggest the dynamic behavior of the 6-MePyS ligand, which has already been observed.^33^ It is unclear whether the compound derives from the decoordination of inserted acetylene from complex **6b** or is directly formed from starting compound **1** under substituting acetylene.

**Scheme 5:**
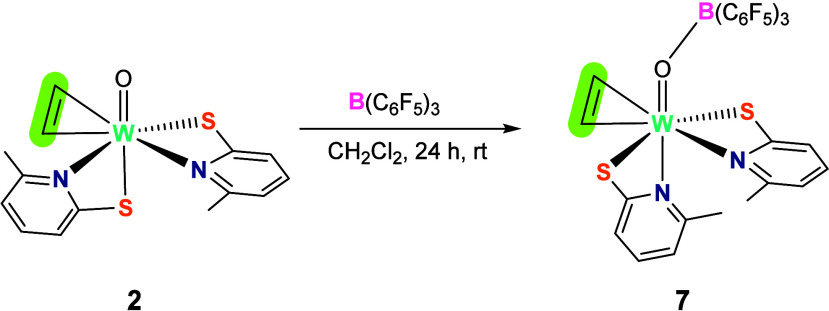
Formation of the Lewis Adduct between Tungsten Oxido Complex (**2**) and B(C_6_F_5_)_3_

Since the isocyanide exhibited no reactivity with **2**, we opted to enhance the electrophilicity of the tungsten oxido complex by the introduction of a Lewis acid. Thus, portionwise addition of tris(pentafluorophenyl)borane (B(C_6_F_5_)_3_) in CH_2_Cl_2_ to a solution of [WO(C_2_H_2_)(6-MePyS)_2_] (**2**) in the same solvent caused a color change from light orange to dark red upon stirring for 24 h. After solvent removal, the solids were washed with heptane to yield 76% of borane adduct [W{OB(C_6_F_5_)_3_}(C_2_H_2_)(6-MePyS)_2_] (**7**) as an orange powder (Scheme 5). Product **7** is highly soluble in dichloromethane and toluene, whereas it is insoluble in heptane and pentane.

**Figure 5:**
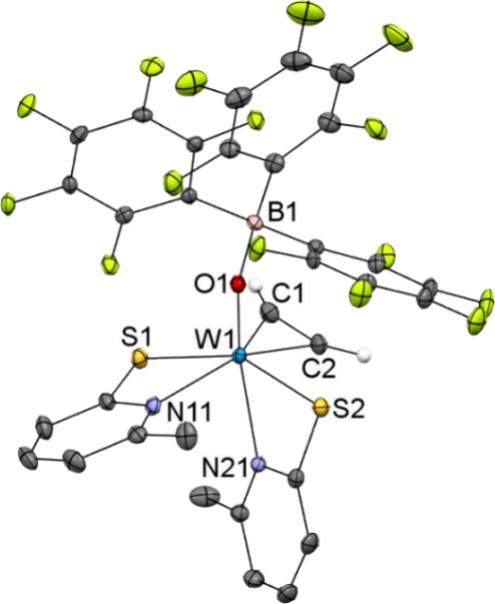
Molecular structure of [W{OB(C_6_F_5_)_3_}(C_2_H_2_)(6-MePyS)_2_] (**7**) showing the atomic numbering scheme. The ellipsoids are drawn at the 50% probability level. The H atoms were omitted for clarity, except for those at the acetylene.

The adduct was crystallized from CH_2_Cl_2_/heptane upon cooling to −37 °C, allowing single crystal X–ray diffraction analysis, which revealed B(C_6_F_5_)_3_ bound to the oxido ligand of starting compound **2**. The molecular structure is listed in Figure 5. Interestingly, the starting compound underwent a conformational change upon adduct formation with B(C_6_F_5_)_3_ from the *S*,*S*-*cis* to the *S*,*S*-*trans* geometry. Notably, the W–S bonds are, however, not equally long [W1–S1 2.3711(9) Å, W1–S2 2.5160(9) Å, S1–W1–S2 151.35(3)°]. The significant difference can be reasoned via the elongation of the W1–N11 bond due to the *trans* position of the acetylene ligand, which indirectly causes the shortening of the W1–S1 bond. As for the starting compound **2** [C1–C2 1.279(2) Å, W1–C_2_ 1.9849(17) Å],^19^ the acetylene ligand in **7** [C1–C2 1.278(5) Å, W1–C_2_ 1.957(3) Å] is almost normal to the W=O bond [C1–C2–W1–O1 82.6(3)°, C2–C1–W1–O1 105.6(3)°]. Due to the presence of B(C_6_F_5_)_3_, the W1–O1 bond is only slightly elongated [W1–O1 1.771(3) Å for **7** and W1–O1 1.7153(13) Å for **2**] and the O1–B1 bond [1.518(5) Å] and W1–O1–B1 angle [165.8(2)°] are similar to the already reported Mo imido oxido B(C_6_F_5_)_3_ adduct [O2–B1 1.5002(16) Å, Mo1–O2–B1 162.72(9)°]^48^ or Mo dioxido B(C_6_F_5_)_3_ adduct [O2–B1 1.530(2) Å, Mo1–O2–B1 159.08(9)°].^49^

The compound is not stable in solution at rt, which prevented obtaining completely pure ^1^H NMR spectra. Nonetheless, by crystallization at −37 °C, a sample was isolated in pure form, as validated by elemental analysis. ^1^H NMR spectroscopy shows a downfield shift of acetylenic protons from 11.23 and 10.99 ppm (complex **2**)^19^ to 12.21 and 12.01 ppm due to a more electron-deficient tungsten center. The coordination of the Lewis acid to the oxido group is further confirmed via ^19^F NMR spectroscopy as indicated by the shifting of the resonances from uncoordinated borane (−128.14 ppm, −143.81 ppm, −160.95 ppm)^50^ to coordinated borane (−133.01 ppm, −159.59 ppm, −165.18 ppm). The W–O–B adduct is highly sensitive toward water, forming known hydroxy borate anion [HO-B(C_6_F_5_)_3_]^−^^51^ and a decomposed complex species. Coordinating solvents can cleave the borane–oxygen bond to form [solvent···B(C_6_F_5_)_3_] adducts instead, and the parent complex is regained, as observed in acetonitrile. This behavior limits the scope of the appropriate solvents when handling the W–O–B adduct. Unfortunately, additions of nucleophiles such as PMe_3_ or ^*t*^BuNC lead to borane abstraction and the formation of [Me_3_P···B(C_6_F_5_)_3_] or [^*t*^BuNC···B(C_6_F_5_)_3_] adducts and the starting oxido complex **2**.

The reactions of complexes [W(CO)(C_2_H_2_)(6-MePyS)_2_] (**1)** and [WO(C_2_H_2_)(6-MePyS)_2_] (**2**) with nucleophiles are significantly different depending on the nucleophile used (PMe_3_ vs ^*t*^BuNC). As mentioned above, in the reaction of W(II) complex **1** and excess phosphine, PMe_3_ is capable of attacking the acetylenic carbon, thereby, forming an ionic W carbyne species. In the case of excess of ^*t*^BuNC, the CO ligand in **1** is initially replaced by isocyanide, as the two ligands are isolobal and have π-acceptor abilities,^52^ contrasting the analogous reaction with PMe_3_, where the CO ligand remains coordinated.^19^ Replacement of the carbonyl ligand in **1** by isocyanide destabilized the acetylene ligand as observed by variable temperature NMR spectra (Figure 3). In general, isocyanide ligands are weaker π-acceptors than carbonyls.^53,54^ Two rotamers can be envisioned upon rotation of the acetylene by 90°, in which different sets of orbitals are used. Thereby, the nature of the orbital overlap swaps between σ- and π-interactions. Thus, in a complex coordinated by the weaker π-acceptor isocyanide, the energy difference of the two rotamers is expected to be smaller, resulting in a lower barrier as observed here. Upon coordination of a second isocyanide, this trend increases (acetylene signal splitting observed in **4** at −10 °C and in **5** at −20 °C). Insertion of acetylene into the W–N bond, as found in **6a**+**6b**, occurs after the addition of three or more equiv of ^*t*^BuNC. This is likely due to steric crowding, a common strategy used to enhance the insertion rate.^55^ Thus, regardless of the nucleophile (PMe_3_ or isocyanides), the tungsten center is the preferred target for the attack in studied acetylene complexes. However, the more π-acidic character of isocyanide compared to PMe_3_^56^ renders the former a weaker nucleophile to attack acetylene, preventing carbyne formation as observed with PMe_3_. In the isocyanide reaction, the neighboring N atom of the ancillary ligand appears to be a stronger nucleophile as acetylene inserts into the W–N bond (**6a**+**6b**). In addition, the weaker capability for a nucleophilic attack of ^*t*^BuNC prevents coordination to the tungsten(IV) complex **2** in contrast to PMe_3_ where a phosphine-stabilized vinyl species is obtained (Scheme 1). The introduction of the borane to compound **2** did not result in the desired activation of coordinated acetylene. Instead, reactions with nucleophiles with the adduct **7** led to the recovery of the initial complex **2**. Interestingly, Lewis adduct formation between a borane and a tungsten oxido complex bearing π-bound ligand is rare and has only previously been suggested by NMR spectroscopy.^57^

Although isocyanide is not a biological nucleophile, the reactivity observed delivers interesting information about the behavior of the potential acetylene adduct in the enzyme. As coordination of isocyanide molecules to W caused insertion of the acetylene, such insertion could also be envisioned into a W–S bond in the active site due to the steric constraint caused by the coordination environment. A possible candidate could be Cys141 in the active site of the enzyme (Figure 1). Although unlikely for electronic reasons, an alternative scenario where direct hydration of the coordinated acetylene occurs within the enzyme can still be envisioned; however, such a reactivity would be completely controlled by steric factors in the active site.

## Conclusion

Two sulfur-rich tungsten acetylene complexes **1** and **2** with different metal–acetylene binding (limiting cases according to the Dewar–Chatt–Duncanson model) were chosen to mimic the organometallic intermediate suggested for the enzyme AH. According to previous computational studies, acetylene coordination is followed by water or hydroxide nucleophilic attack on acetylenic carbon.^58^ Our current and previous results suggest that such a reaction is not possible for the tungsten-coordinated acetylene due to the high electrophilicity of the tungsten center. The reaction of complex **2** (with tungsten in the biological oxidation state IV) with an aqueous base led to the thermodynamically driven oxygen atom transfer from water to the W(IV) center. Coordinated acetylene acts as a two-proton-two-electron acceptor and is reduced to ethylene. Such a reactivity demonstrates the possible use of tungsten(IV) alkyne complexes as synthons for vicinal olefin dianions in organic synthesis. The fact that tungsten(II) carbonyl complex **1** does not react with the aqueous base points toward the requirement for d^2^ metal center, which was previously reported for substituted alkyne Nb(III) and Ta(III) complexes.^35,36^ Although not activated in the desired fashion, acetylene in **2** undergoes nitrogenase-like reduction to ethylene. Acetylene reduction assay is a commonly used method for determining the activity of the isolated nitrogenases.^59^ For nitrogenase, a mechanism that involves an enzyme-bound iron η^2^-vinyl intermediate is suggested,^60^ contrasting the here suggested requirement for a d^2^ metal center. On the other hand, vinylation of acetylene can occur with the electron-richer complex **1** under increased steric bulk caused by the incoming nucleophiles. Such intramolecular nucleophilic attack occurred upon adding an excess of the bulky nonbiological nucleophile *tert*-butyl isocyanide. Initially, the isocyanide binds to the electrophilic W(II) center, a reactivity previously observed with PMe_3_.^19^ Finally, to indirectly activate W-bound acetylene and simulate the potential interaction of W=O with a second coordination sphere, B(C_6_F_5_)_3_ was added as a Lewis acid. However, upon the addition of nucleophiles, the Lewis acid gets detached from the adduct, contrasting the desired attack on the coordination alkyne or the previously observed addition to the metal center.

Most probably, the first shell mechanism suggested for AH by Himo^3^ is unlikely as our extensive studies with nucleophiles, as well as the lack of other examples from the literature, demonstrate the uncertainty of a direct nucleophilic attack of water on coordinated acetylene.

## Experimental Section

All experimental details are described in the Supporting Information file. Safety statements:

*CAUTION!* Extreme care should be taken in handling cryogen liquid nitrogen and its use in the Schlenk line trap to avoid the condensation of oxygen from the air.

*CAUTION!* Acetylene is a highly flammable and explosive gas. It was always used in a well-ventilated fume hood and kept away from the heat sources.

*CAUTION! tert*-Butyl isocyanide is a flammable liquid with acute inhalation toxicity. It should be handled with care. All manipulations were performed on the smallest possible scale.

## Supporting Information Available

Full experimental procedures, NMR spectra, and crystallographic data (PDF)
