# Supplementary material for: Nucleophiles Target the Tungsten Center Over Acetylene in Biomimetic Models
Source: Inorg Chem. 2024 Jun 14;63(26):11953–62. doi: 10.1021/acs.inorgchem.4c00286 (PMC11220757; doi:10.1021/acs.inorgchem.4c00286)
Supplement: Supplementary file 1 — ic4c00286_si_001.pdf [file ic4c00286_si_001.pdf]

# Supporting Information

## Nucleophiles Target the Tungsten Center Over Acetylene in Biomimetic Models

*Miljan Z. Ćorović,* [‡, a] *Angela Milinkovic,* [‡, a] *Niklas Stix,* [a] *Antoine Dupé,* [a] *Nadia C. Mösch-Zanetti** [, a]

[a] *Institute of Chemistry, Inorganic Chemistry, University of Graz, 8010 Graz, Austria*

‡These authors contributed equally.

* Corresponding author. Tel.: +43 (0)316 380 – 5286

E-mail address: nadia.moesch@uni-graz.at

## Table of Contents

1 General Considerations ........ S1

2 Syntheses ........ S2

3 Reactivity Studies ........ S9

4 NMR Spectra ........ S13

5 IR Spectrum ........ S35

6 Line Shape Analysis ........ S36

7 Crystal Structure Determination ........ S37

8 References ........ S45

# 1 General Considerations

All experiments were carried out under inert atmosphere employing standard Schlenk and glovebox techniques unless otherwise stated. Commercially available chemicals were used as received. Air and moisture sensitive chemicals were stored in Schlenk flasks or under $N_2$ atmosphere in a glovebox; liquids were additionally stored over molecular sieves. The tungsten complexes $WO(C_2H_2)(6\text{-}MePyS)_2$, $W(CO)(C_2H_2)(6\text{-}MePyS)_2$,[1] $WO(HC_2Ph)(6\text{-}MePyS)_2$,[2] and the Lewis acid $B(C_6F_5)_3$[3] were prepared according to published procedures using Schlenk and glovebox techniques. NaOD solution was prepared by dissolving sodium in $D_2O$. Oxygen-free solutions of NaOH were obtained by flushing the solution with $N_2$ for 1 h. The solution of NaOD in $D_2O$ was prepared by dissolving elemental Na in $D_2O$. All solvents were purified by a Pure Solv Solvent Purification System and stored over activated molecular sieves (3  or 4 Å). NMR spectra were recorded using Bruker Avance III and Bruker Avance NEO 500 MHz spectrometers. $^{1}H$ NMR spectra were recorded at 300 MHz for room temperature or at 500 MHz for low-temperature measurements and referenced to residual protons of the NMR solvents. $^{13}C$ NMR spectra were obtained at 75 MHz and spectra were referenced to the deuterated solvent peak. $^{11}B$ NMR spectra were obtained at 128 MHz and shifts are referenced to external standard $BF_3{\cdot}Et_2O$. $^{19}F$ NMR spectra were obtained at 282 MHz and shifts are referenced to external standard $CFCl_3$. The chemical shifts δ are given in ppm. The multiplicity of peaks is denoted as broad singlet (bs), singlet (s), doublet (d), triplet (t), quadruplet (q), multiplet (m). Coupling constants *J* are given in Hertz. Mass spectroscopy measurements using electron impact ionization (EI-MS) have been performed with an Agilent 5973 MSD with a push rod for direct sample measurement. IR spectra were recorded in the solid-state at a resolution of 2 $cm^{-1}$ on a Bruker ALPHA-P Diamant ATR-FTIR. IR spectra of gaseous samples were measured at a resolution of 4 $cm^{-1}$ on Lyza 7000 spectrometer (Anton Paar). Elemental analyses (C, H, N)

were carried out by the Department of Inorganic Chemistry at the Graz University of Technology (Heraeus Vario Elementar automatic analyzer).

**Safety statements**:

*CAUTION!* Extreme care should be taken both in the handling of the cryogen liquid nitrogen and its use in the Schlenk line trap to avoid the condensation of oxygen from the air.

*CAUTION!* Acetylene is a highly flammable and explosive gas. It was always used in the well-ventilated fume hood and kept away from heat sources.

*CAUTION! Tert*-butyl isocyanide is a flammable liquid with acute inhalation toxicity. It must be handled with care. All manipulations were performed on the smallest possible scale.

# 2 Syntheses

**[W(CN$^t$Bu)($C_2H_2$)(6-MePyS)$_2$]** (**4**) + **[W(CN$^t$Bu)$_2$($C_2H_2$)(6-MePyS)(*S*−6-MePyS)]** (**5**)**.** The purple complex [W(CO)($C_2H_2$)(6-MePyS)$_2$] (**1**) (100 mg, 0.20 mmol, 1.0 equiv) was dissolved in 5 mL $CH_2Cl_2$ and 2.0 equiv *tert*-butyl isocyanide (45 µL, 0.40 mmol) in 2 mL $CH_2Cl_2$ was added. Within 5-10 min a color change from purple to dark red and then to brown occurred. After 6 h of stirring at room temperature, the reaction solution was evaporated to dryness. Diethyl ether was added to the brown powder, whereupon a brown solution and a red-brown precipitate is obtained. The ether was removed via cannula filtration and the filtrate is stored at -37 °C for 24 h. Subsequently, product [W(CN$^t$Bu)$_2$($C_2H_2$)(6-MePyS)(*S*−6-MePyS)] (**5**) precipitated as brown crystals and were isolated via cannula filtration. Compound **5** is further washed with cold ether (3x 5 mL) and pentane (3x 5 mL). After drying product **5** in vacuo, a brown powder in 48% yield (60 mg) is obtained. Single crystals suitable for X-ray diffraction analysis were obtained from a concentrated ether solution at -37 °C. The collected yellow ether fractions from the cannula filtrations were evaporated to obtain the mono-isocyanide product

$[W(CN^tBu)(C_2H_2)(6\text{-MePyS})_2]$ (**4**) from this filtrate. Product **4** is washed with pentane (3x 3 mL) to give $[W(CN^tBu)(C_2H_2)(6\text{-MePyS})_2]$ as a light-brown powder in low yield (<20%).

Mono-isocyanide product **$[W(CN^tBu)(C_2H_2)(6\text{-MePyS})_2]$** (**4**) $^1H$ NMR (300 MHz, $CD_2Cl_2$) δ 13.42 (s, 2H, HC≡CH), 7.48 (t, *J* = 7.8 Hz, 1H, pyH-*p*), 6.86 (dt, *J* = 7.7, 3.9 Hz, 2H, pyH-*p*, pyH-*m*), 6.65 (d, *J* = 8.0 Hz, 1H, pyH-*m*), 6.52 (d, *J* = 7.7 Hz, 2H, pyH-*m*), 1.90 (s, 3H, $CH_3$), 1.45 (s, 9H, $^tBu$), 1.14 (s, 3H, $CH_3$). $^{13}C$ NMR (75 MHz, $CD_2Cl_2$, W-C≡N and C≡C obscured) δ 179.60 (pyC-*o*), 169.97 (pyC-*o*), 158.49 (pyC-*o*), 154.03 (pyC-*o*), 137.87 (pyC-*p*), 134.28 (pyC-*p*), 124.31 (pyC-*m*), 123.78 (pyC-*m*), 119.25 (pyC-*m*), 117.58 (pyC-*m*), 63.09 ($qCMe_3$), 32.21 ($^tBu$), 31.32 ($^tBu$), 26.88 ($CH_3$), 21.98 ($CH_3$). IR ($cm^{-1}$): 3687(w), 2970 (m), 2928 (m), 2867 (m), 2126 (m), 2020 (m, C≡N), 1908 (s, C≡N), 1586 (m), 1549 (s), 1488 (m), 1447 (s), 1429 (s), 1192 (s), 1170 (s), 937 (m), 876 (m), 764 (s), 725 (s), 680 (m), 569 (m), 560 (m), 524 (m), 430 (m). EI-MS (70 eV) *m/z*: $[M]^+$ 541.2. Anal. Calcd. for $C_{19}H_{23}N_3S_2W$: C, 42.15; H, 4.46; N, 7.75. Found: C, 42.43; H, 4.53; N, 7.76.

Bis-isocyanide product **$[W(CN^tBu)_2(C_2H_2)(6\text{-MePyS})(S\text{−}6\text{-MePyS})]$** (**5**) $^1H$ NMR (300 MHz, $CD_2Cl_2$) δ 12.61 (s, 2H, HC≡CH), 7.65 – 6.21 (bs, m, 6H, pyH), 2.39 (bs, 3H, $CH_3$), 1.37 (s, 18H, 2x $^tBu$). $^{13}C$ NMR (75 MHz, $CD_2Cl_2$, W-C≡N obscured) δ 195.81 (C≡C), 155.48 (pyC-*o*), 134.80 (pyC-*p*), 124.03 (pyC-*m*), 116.63 (pyC-*m*), 57.53 ($qCMe_3$), 31.31 ($^tBu$), 26.83 ($CH_3$). IR ($cm^{-1}$): 3052(w), 2979 (m), 2128 (m, C≡N), 2036 (s, C≡N), 1571 (m), 1550 (s), 1505 (m), 1449 (m), 1425 (s), 1367 (m), 1231 (m), 1194 (s), 1175 (s), 1152 (s), 1134 (m), 1081 (m), 998 (m) 765 (s), 705 (s), 526 (s), 465 (s). EI-MS (70 eV) *m/z*: $[M\text{-}(CN^tBu)_2]^+$ 458.1. Anal. Calcd. for $C_{24}H_{32}N_4S_2W\cdot 0.4\ C_4H_{10}O$: C, 47.00; H, 5.54; N, 8.57. Found: C, 47.07; H, 5.17; N, 8.97.

For the **VT-NMR experiment** reaction conditions were chosen as follows: 1.0 equiv $W(CO)(C_2H_2)(6\text{-MePyS})_2$ (20 mg, 0.04 mmol) and 2.0 equiv *tert*-butyl isocyanide (9 µL, 0.08 mmol) in $CD_2Cl_2$ were kept at rt for 18 h.

**[W(*C*,*S*−CHCH−*N*−6-MePyS)(CN$^t$Bu)$_5$][Cl]** (**6a**) + **[W(*C*,*S*−CHCH−*N*−6-MePyS)(*S*−6-MePyS)(CN$^t$Bu)$_4$]** (**6b**). The purple complex [W(CO)($C_2H_2$)(6-MePyS)$_2$] (**1**) (60 mg, 0.12 mmol, 1.0 equiv) was dissolved in 3 mL $CH_2Cl_2$ and 10.0 equiv *tert*-butyl isocyanide (135 µL, 1.2 mmol) in 3 mL $CH_2Cl_2$ was added dropwise. Within 5-10 min a color change from purple to brown-red and then to cherry-red occurred. After 8 h of stirring at room temperature, the reaction solution was evaporated to dryness. Diethyl ether was added to the red-brown wax whereupon a red-orange solution and a brown precipitate was obtained. The ether was removed via cannula filtration and the residue was further washed with cold ether (4-5 x 3 mL) until the washing solution turned colorless. Additionally, the product was washed twice with toluene (3 mL) and pentane (2 x 3 mL). Subsequently, the product is dried in vacuo to give a red powder (50 mg).

Major compound **[W(*C*,*S*−CHCH−*N*−6-MePyS)(CN$^t$Bu)$_5$][Cl]** (**6a**) $^1$H NMR (300 MHz, $CD_2Cl_2$) δ 8.53 (d, *J* = 15.3 Hz, 1H, HC=CH), 7.55 (t, *J* = 7.7 Hz, 1H, pyH-*p*), 7.18 – 7.11 (m, 2H, HC=CH, pyH-*m*), 7.05 (d, *J* = 7.6 Hz, 1H, pyH-*m*), 2.53 (s, 3H, $CH_3$), 1.42 (s, 9H, $^t$Bu), 1.38 (d, *J* = 2.5 Hz, 36H, 4x $^t$Bu). $^{13}$C NMR (75 MHz, $CD_2Cl_2$) δ 186.19 (pyC-*o*), 159.57 (pyC-*o*), 153.30 (W-C≡N), 142.71 (HC=CH), 137.78 (pyC-*p*), 121.60 (pyC-*m*), 120.63 (pyC-*m*), 115.41 (HC=CH), 62.96 (qCMe$_3$), 60.30 (qCMe$_3$), 57.55 (qCMe$_3$), 31.48 ($^t$Bu), 31.22 ($^t$Bu), 31.14 (2x $^t$Bu), 30.08 ($^t$Bu), 24.70 ($CH_3$).

Minor compound **[W(*C*,*S*−CHCH−*N*−6-MePyS)(*S*−6-MePyS)(CN$^t$Bu)$_4$]** (**6b**)$^1$H NMR (300 MHz, $CD_2Cl_2$) δ 7.93 (d, *J* = 10.6 Hz, 1H, HC=CH), 7.49 (t, *J* = 7.7 Hz, 1H, pyH-*p*), 7.31 (d, *J* = 7.7 Hz, 1H, pyH-*m*), 6.98 (d, *J* = 7.6 Hz, 1H, pyH-*m*), 6.70 (t, *J* = 7.0 Hz, 1H, pyH-*p*), 6.61 (d, *J* = 10.6 Hz, 1H, HC=CH), 6.46 (d, *J* = 8.1 Hz, pyH-*m*), 2.41 (s, 2H, $CH_3$), 2.34 (s, 2H, $CH_3$), 1.47 (s, 27H, 3x $^t$Bu), 1.37 (s, 9H, $^t$Bu). $^{13}$C NMR (75 MHz, $CD_2Cl_2$) δ 179.06 (pyC-*o*), 157.79 (pyC-*o*), 138.34 (pyC-*p*), 119.36 (pyC-*m*), 116.79 (HC=CH), 57.32 (qCMe$_3$), 31.14 ($^t$Bu), 29.65 ($^t$Bu).

IR ($cm^{-1}$) **6a**+**6b**: 2973 (m), 2091 (s, C≡N), 2033 (s, C≡N), 1847 (m, C≡N), 1688 (w), 1589 (w), 1437 (w), 1366 (m), 1188 (s), 588 (w), 530 (m), 428 (m).

Data of side product **[6-MePySCD$_2$Cl]** are in accordance with literature.[1] $^1$H NMR (300 MHz, $CD_2Cl_2$) δ 7.49 (t, *J* = 7.7 Hz, 1H, pyH-*p*), 7.07 (d, *J* = 7.8 Hz, 1H, pyH-*m*), 6.99 (d, *J* = 8.0 Hz, 1H, pyH-*m*), 2.53 (s, 3H, $CH_3$).

**[W(CN$^t$Bu)$_4$(6-MePyS)(*S*−6-MePyS)]** (**6c**). The washing fractions (ether, toluene and pentane) from the reaction of **6a** + **6b** were collected in a vial, whereupon after 24 h a red precipitate had formed. The solvents were removed and after drying in vacuo, the residue was dissolved in a $CH_3CN$/heptane solvent mixture. Storage at -37 °C overnight led to red single crystals suitable for X-ray diffraction analysis. $^1$H NMR (300 MHz, $CD_3CN$) δ 7.17 (t, *J* = 7.7 Hz, 2H), 6.92 (d, *J* = 8.0 Hz, 2H), 6.64 (d, *J* = 7.7 Hz, 2H), 2.37 (s, 6H), 1.39 (s, 36H). $^{13}$C NMR (75 MHz, $CD_3CN$, pyC-*o* and W-C are obscured) δ 157.10 (pyC-*o*), 134.96 (pyC-*p*), 126.72 (pyC-*m*), 116.61 (pyC-*m*), 58.71 (qCMe$_3$), 32.27 ($^t$Bu), 31.95 ($^t$Bu), 31.31 ($^t$Bu), 24.44 ($CH_3$).

**[W{O(B(C$_6$F$_5$)$_3$)}(C$_2$H$_2$)(6-MePyS)$_2$]** **(7).** Tris(pentafluorophenyl)borane (270 mg, 0.53 mmol, 1.0 equiv) was dissolved in 10 mL $CH_2Cl_2$ and 1.0 equiv WO($C_2H_2$)(6-MePyS)$_2$ (250 mg, 0.53 mmol) was added portionwise whereupon the reaction color turned from light orange to dark red. After stirring for 24 h at room temperature $CH_2Cl_2$ was evaporated and the obtained red crystalline product was washed with heptane (3x 5 mL), pentane (3x 5 mL) and dried in vacuo to give the adduct as an orange powder in good yield (400 mg, 76%). Single crystals suitable for X-ray diffraction analysis were obtained via crystallization from a concentrated $CH_2Cl_2$/heptane mixture at -37 °C. $^1$H NMR (300 MHz, $CD_2Cl_2$) δ 12.21 (s, 1H, C≡CH), 12.01 (s, 1H, C≡CH), 7.72 (t, *J* = 7.9 Hz, 1H, pyH-*p*), 7.29 (t, *J* = 7.8 Hz, 1H, pyH-*p*), 7.07 (d, *J* = 7.9 Hz, 1H, pyH-*m*), 7.01 (d, *J* = 7.9 Hz, 1H, pyH-*m*), 6.88 (d, *J* = 8.1 Hz, 1H, pyH-*m*), 6.49 (d, *J* = 7.5 Hz, 1H, pyH-*m*), 2.80 (s, 3H, $CH_3$), 1.86 (s, 3H, $CH_3$). $^{13}$C NMR (75 MHz,

$CD_2Cl_2$) δ 179.59 (pyC-*o*), 176.61 (pyC-*o*), 174.92 (C≡CH), 169.80 (C≡CH), 161.21 (pyC-*o*), 155.95 (pyC-*o*), 149.47 ($CF_3$), 146.14 ($CF_3$), 140.90 (pyC-*p*), 138.87 ($CF_3$), 138.17 (pyC-*p*), 135.63 ($CF_3$), 125.14 (pyC-*m)*, 124.16 (pyC-*m*), 124.08 (pyC-*m*), 119.13 (pyC-*m*), 25.14 ($CH_3$), 24.17 ($CH_3$). $^{19}F$ NMR (471 MHz, $CD_2Cl_2$) δ -133.01 (d, *J* = 26.6 Hz, 6F, *o*-F), -159.59 (t, *J* = 20.3 Hz, 3F, *p*-F), -165.18 – -165.63 (m,6F, *m*-F). IR ($cm^{-1}$): 1644 (w), 1593 (w), 1515 (m), 1455 (s), 1378 (w), 1280 (w), 1175 (w), 1087 (s), 973 (s, W=O), 773 (m), 657 (s), 633 (s), 575 (m), 444 (w). EI-MS (70 eV) *m/z*: $[M-(C_2H_2)-(B(C_6F_5)_3)]^+$ 448.0, $[M-(C_2H_2)-(6\text{-}MePyS)]^+$ 836.3. Anal. Calcd. for $C_{32}H_{14}BF_{15}N_2OS_2W$: C, 38.97; H, 1.43; N, 2.84. Found: C, 38.63; H, 1.44; N, 2.87.

**Table S1.** Relevant $^{1}H$ NMR, $^{13}C$ NMR and IR data of W(II) species with isocyanides. [a)] Data given in $CD_3CN$, n.d. = not detected

| | HC≡CH or HC═CH | | $CH_3$ (s) | | *para* pyH (t)/pyC | | C≡O | | C≡N | |
|---|---|---|---|---|---|---|---|---|---|---|
| NMR in $CD_2Cl_2$ [ppm]<br>IR ν[cm$^{-1}$] | $^{1}H$ | $^{13}C$ | $^{1}H$ | $^{13}C$ | $^{1}H$ | $^{13}C$ | $^{13}C$ | IR | $^{13}C$ | IR |
| 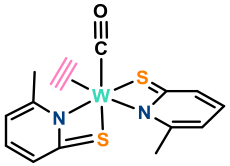<br>**1** | 13.77<br>12.50 | 205.73<br>204.14 | 1.91<br>1.22 | 26.88<br>22.08 | 7.55<br>7.04 | 138.77<br>135.97 | 237.09 | 1891<br>1854 | - | - |
| 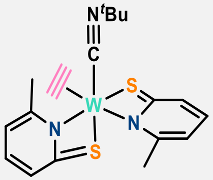<br>**4** | 13.42 | n.d. | 1.90<br>1.14 | 26.88<br>21.98 | 7.48<br>6.86 | 137.87<br>134.28 | - | - | n.d. | 2020<br>1908 |
| 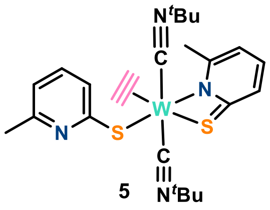<br>**5** | 12.61 | 195.81 | 2.39 | 26.83 | | 134.80 | - | - | n.d. | 2128<br>2036 |

| | | | | | | | | | | |
|---|---|---|---|---|---|---|---|---|---|---|
| 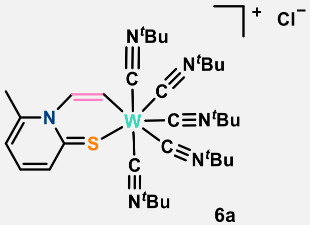 6a | 8.53 | 142.71 | 2.53 | 24.70 | 7.55 | 137.78 | - | - | 153.30 | 2091 |
| | 7.18 – 7.11 | 115.41 | | | | | | | | 2033 |
| | | | | | | | | | | 1847 |
| 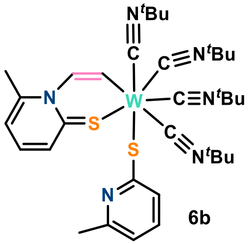 6b | 7.93 | n.d | 2.41 | n.d. | 7.49 | 138.34 | - | - | n.d | 2091 |
| | 6.61 | 116.79 | 2.34 | | 6.70 | n.d. | | | | 2033 |
| | | | | | | | | | | 1847 |
| 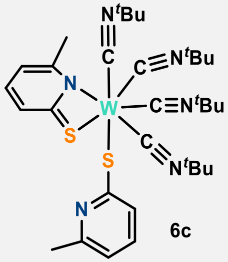 6c | - | - | 2.37[a] | 24.44[a] | 7.17[a] | 134.96[a] | - | - | | |
| 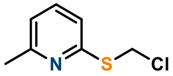 | - | - | 2.53 | | 7.49 | | - | - | - | - |

# 3 Reactivity Studies

**Reactions of complexes 1 and 2 with NaOH/$H_2O$**: Solutions of complexes **1** and **2** (20 mg in 2 mL $CH_2Cl_2$) were combined with 2 mL of 3.75 M NaOH, the reaction mixture was vigorously stirred for 1 h. IR analysis of the atmosphere above the reaction mixture, showed stretching at 950 $cm^{-1}$ assignable to H-C-H out-of-plane wagging[4] in the case of complex **2** (Figure S1). The rest of the signals visible in the spectrum below belong to $CH_2Cl_2$, as confirmed by the blank experiment. No ethylene was detected in the sample containing complex **1**.

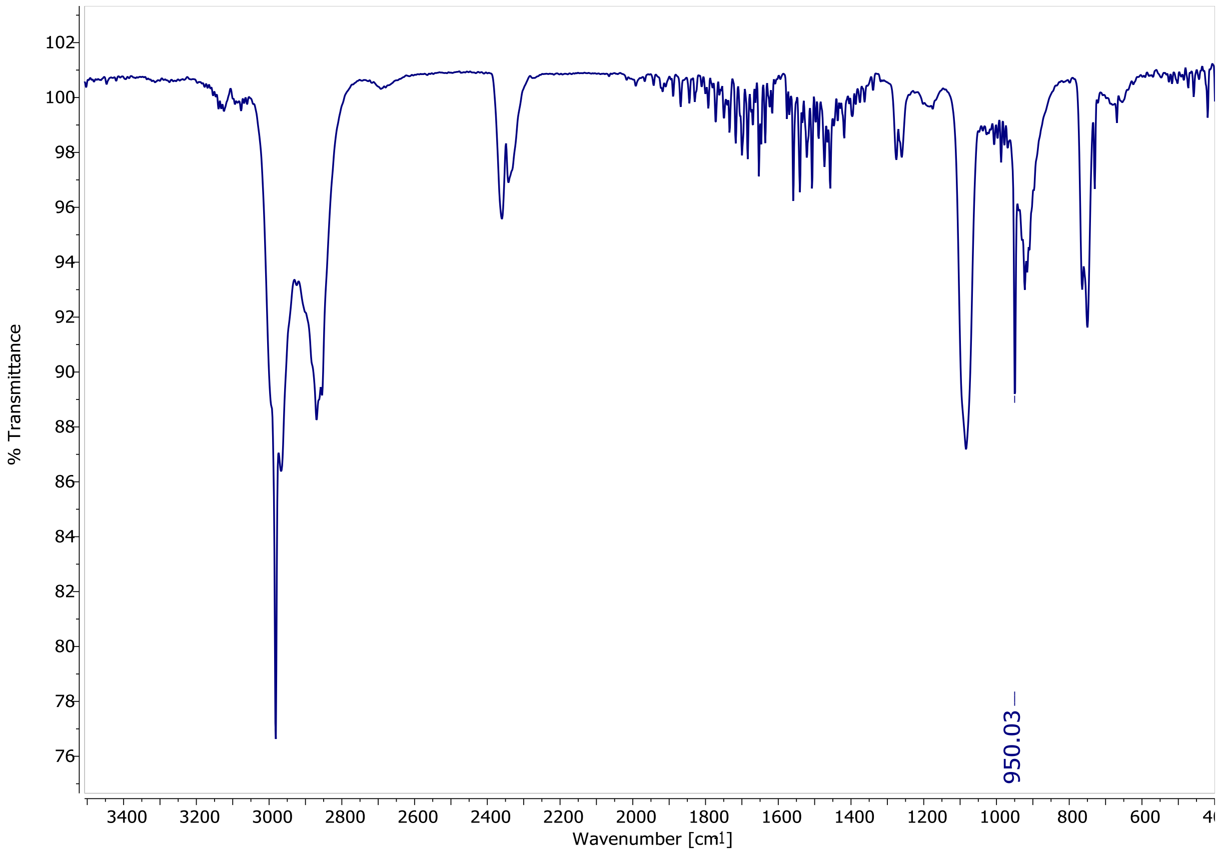


**Figure S1**. IR Spectrum of the atmosphere above the reaction mixture containing complex **2** in $CH_2Cl_2$ and aqueous NaOH solution

**Reactions of complexes 1 and 2 with water**: Solutions of complexes **1** and **2** (40 µmol in 0.5 mL $CD_2Cl_2$) in a J. Young tube were overlayed with 20 µL of distilled water and sonicated for 1 h. Solutions were followed via $^1$H NMR spectroscopy during a couple of days, and partial

decomposition was observed due to protonation of the bidentate ligand to the corresponding thiol 6-MePySH.[5]

**Reactions of complexes 1 and 2 with NaOD/$D_2O$**: Solutions of complexes **1** and **2** (40 μmol in 0.5 mL $CD_2Cl_2$) in a J. Young tube were overlayed with 68.5 μL of 1.2 M NaOD (2.0 equiv) solution in $D_2O$ and sonicated for 1 h. No reaction was observed in the solution containing complex **1**. In the solution containing complex **2**, $^1$H NMR spectroscopy revealed partial decomposition and the formation of semi-deuterated ethylene $CH_2D_2$[6] as shown in the NMR section, Figure S2.

**Reduction of complex 3 with aqueous NaOH solution**: A 25 mL Schlenk tube was charged with WO(6-MePyS)$_2$(HCCPh) (39.6 mg, 1.0 equiv, 83.5 μmol) and dissolved in 3 mL of $CH_2Cl_2$. Mesitylene (10 μL, 0.91 equiv, 75.7 μmol) was then added to the solution as internal standard. Further on, 3 mL of oxygen-free 15% aq. A NaOH solution (excess) was added to the yellow solution and the two-layer reaction mixture was vigorously stirred during 1 h, which caused fading of the organic phase. After the reaction, an aliquot was taken from this $CH_2Cl_2$ layer for GC-MS analysis. Analysis of the mixture revealed quantitative conversion to styrene.

**Intermediate studies via $^1$H NMR spectroscopy:** The stoichiometric addition of NaOH solution was investigated in both $CD_2Cl_2$ and $CDCl_3$, giving consistent results. However, only the spectra obtained in $CDCl_3$ are presented here for clarity.

**NaOH/$H_2O$:** Solution of complex **3** (40 μmol in 0.5 mL $CDCl_3$) in a J. Young tube was overlayed with 22 μL of 3.75 M NaOH (2.0 equiv) solution in $H_2O$ and sonicated for 10 min. $^1$H NMR spectrum was recorded after 1 h (Figure S3), and it shows the presence of starting complex **3**, intermediate Int**1**, styrene and free ligand (Table S2).

**Table S2.** The reaction mixture of the complex **3**, after being mixed with aqueous NaOH solution for 1 h. Aromatic peaks are ommited for clarity.

| **Species** | **Assignable peaks [ppm]** |
|---|---|
| **Complex 3** | Major isomer: 11.18 (s, 1H, $PhC_2$**H**), 2.65 (s, 3H, 6-**Me**PyS), 2.10 (s, 3H, 6-**Me**PyS).<br>Minor isomer: 11.44 (s, 1H, $PhC_2$**H**), 2.45 (s, 3H, 6-**Me**PyS), 2.06 (s, 3H, 6-**Me**PyS). |
| **Int1** | 10.42 (s, 1H, $PhC_2$**H**), 5.57 (d, J = 1.8 Hz, 1H, W-C(Ph)**Ha**Hb), 3.90 (d, J = 1.8 Hz, 1H, W-C(Ph)Ha**Hb**), 2.69 (s, 3H, 6-**Me**PyS). |
| **Styrene** | 6.72 (dd, J = 17.6, 10.9 Hz, 1H, PhC**Ha**CHaHb), 5.75 (d, J = 17.6 Hz, 1H, PhCHaC**Ha**Hb), 5.35 – 5.16 (m, 1H, PhCHaCHa**Hb**). |
| **Free ligand** | 2.42 (s, 3H, 6-**Me**PySH) |

**NaOD/$D_2O$:** A solution of complex **3** (40 µmol in 0.5 mL $CDCl_3$) in a J. Young tube was overlayed with 84 µL of 1.2 M NaOD (2.0 equiv) solution in $D_2O$ and sonicated for 10 min. The $^1$H NMR spectrum was recorded after 1 h (Figure S4) and it shows the presence of starting complex **3**, intermediate Int**1**, styrene and free ligand (Table S3). The reaction was also followed over time showing the increase of styrene peaks, and decrease of Int**1** signals, as shown in Figure S5. Another decreasing signal at 2.23 ppm is assigned to $H_2O$ peak (as confirmed by blank experiment), which disappears due to the slow phase separation.

**Table S3.** The reaction mixture of complex **3**, after mixing with a NaOD/$D_2O$ solution for 1 h. Aromatic peaks are ommited for clarity.

| **Species** | **Assignable peaks [ppm]** |
|---|---|
| **Complex 3** | Major isomer: 11.19 (s, 1H, PhC$_2$**H**), 2.65 (s, 3H, 6-**Me**PyS), 2.10 (s, 3H, 6-**Me**PyS).<br>Minor isomer: 11.44 (s, 1H, PhC$_2$**H**), 2.45 (s, 3H, 6-**Me**PyS), 2.07 (s, 3H, 6-**Me**PyS). |
| **Int1** | 10.42 (s, 1H, PhC$_2$**H**), 5.56 (s, 1H, W-C(Ph)**Ha**D), 2.70 (s, 3H, 6-**Me**PyS). |
| **Styrene** | trans-α,β-$d_2$-styrene, major: 5.30 (m, 1H, PhCDC**H**D)<br>cis-α,β-$d_2$-styrene, minor: 5.73 (m, 1H, PhCDCD**H**) |
| **Free ligand** | 2.43 (s, 3H, 6-**Me**PySH) |

# 4 NMR Spectra

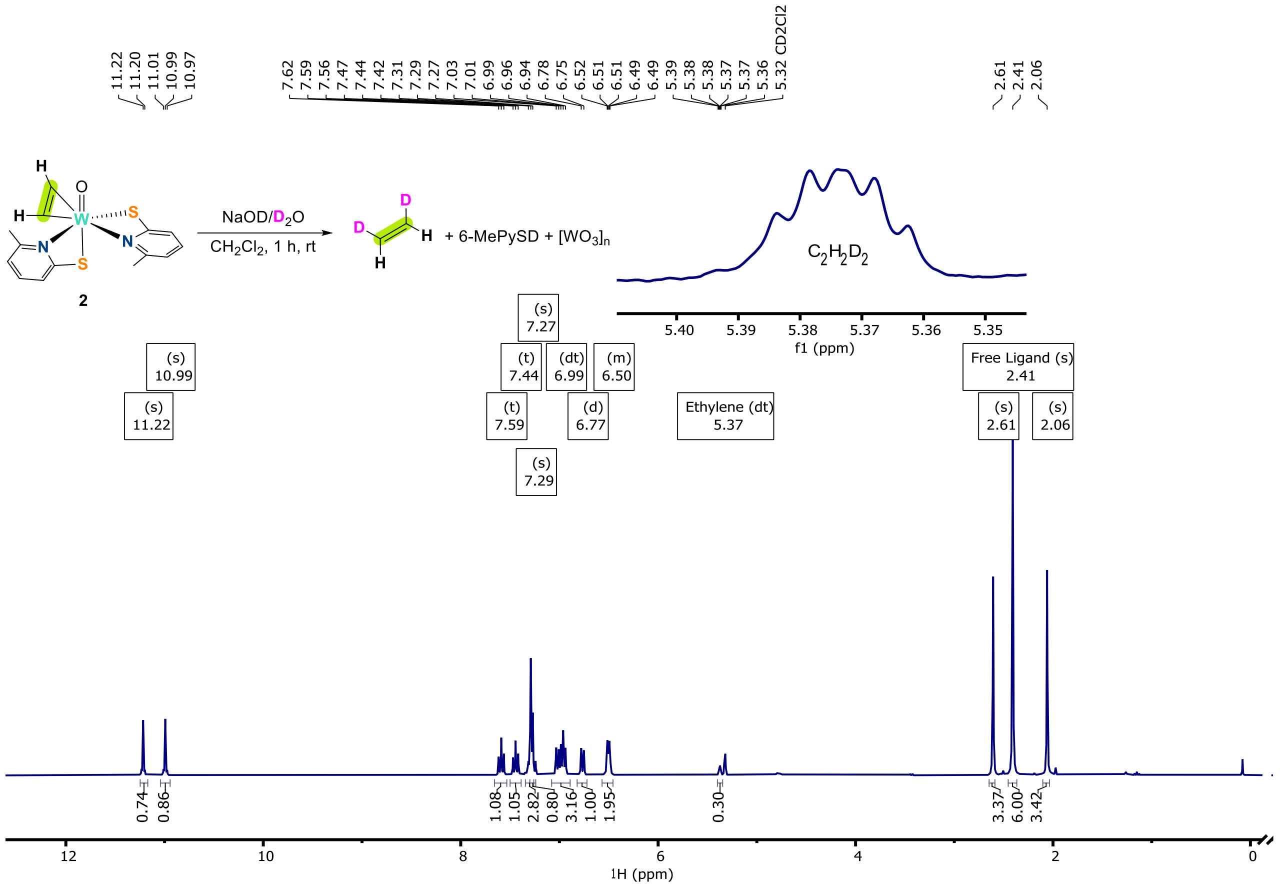


**Figure S2.** $^1$H NMR spectrum of the reaction of complex **2** with 2.0 equiv of NaOD in $CD_2Cl_2/D_2O$ mixture after 1 h. Region between 5.30 – 5.42 ppm is enlarged for better visualization of the $C_2H_2D_2$ peak.

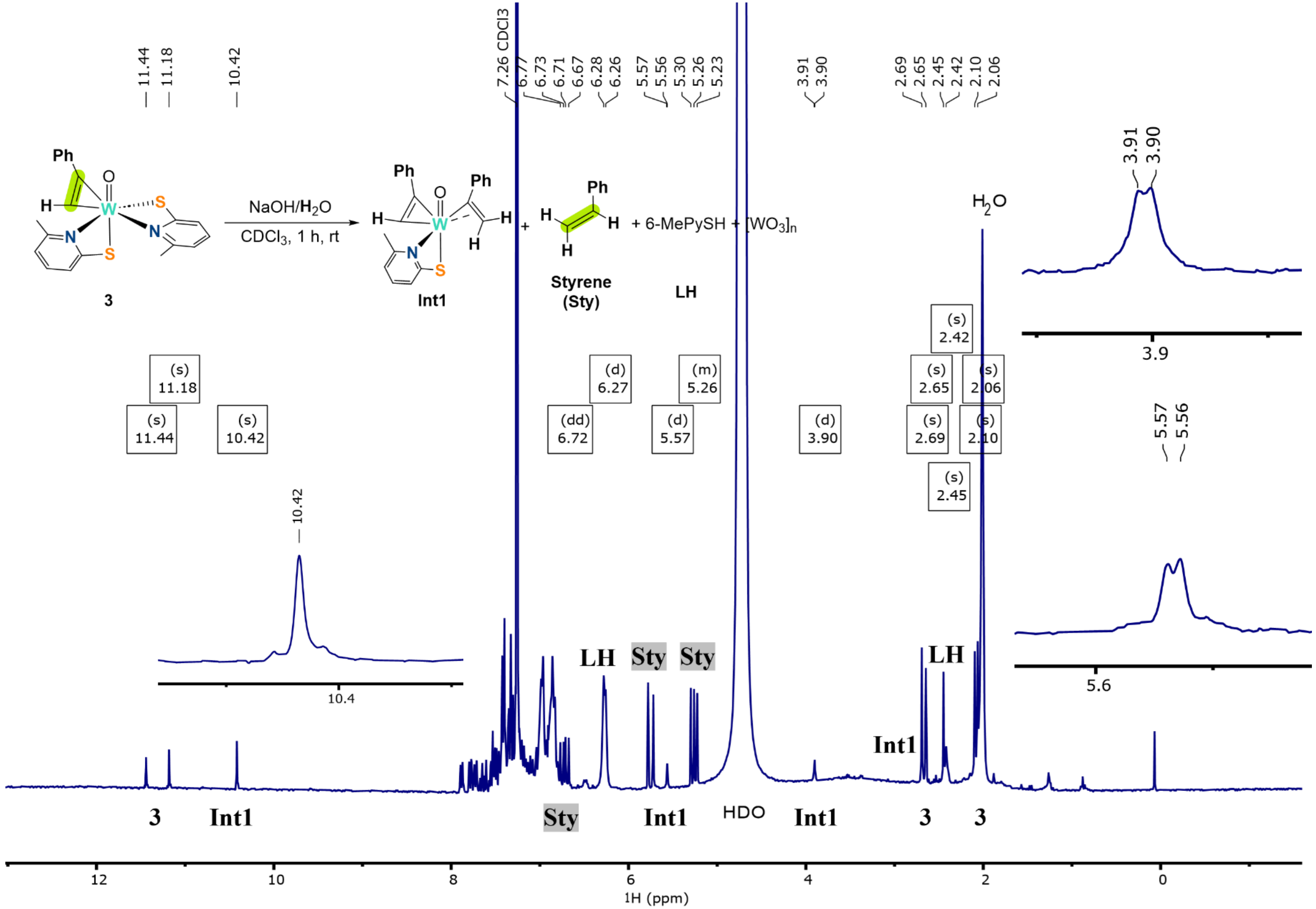


**Figure S3.** $^{1}$H NMR spectrum of the reaction of complex **3** with 2.0 equiv of NaOH in $CDCl_3/H_2O$ mixture after 1 h showing the formation of Int**1**, styrene (**Sty**) and free ligand (**LH**).

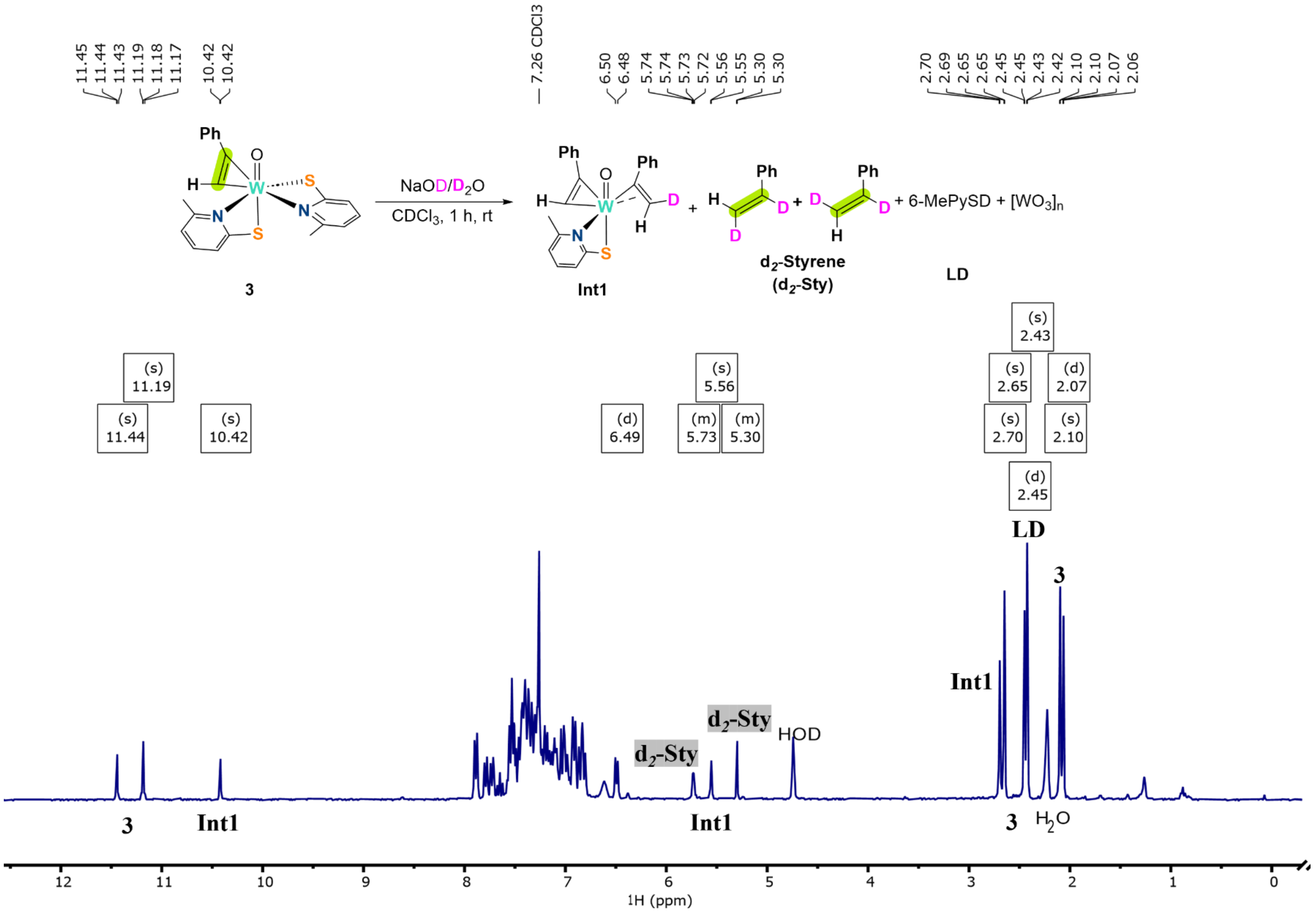


**Figure S4.** $^{1}$H NMR spectrum of the reaction of complex **3** with 2.0 equiv of NaOD in $CDCl_3/D_2O$ mixture after 1 h showing the formation of Int**1**, two d$_2$-styrene isomers (**d$_2$-Sty**) and free ligand (**LD**).

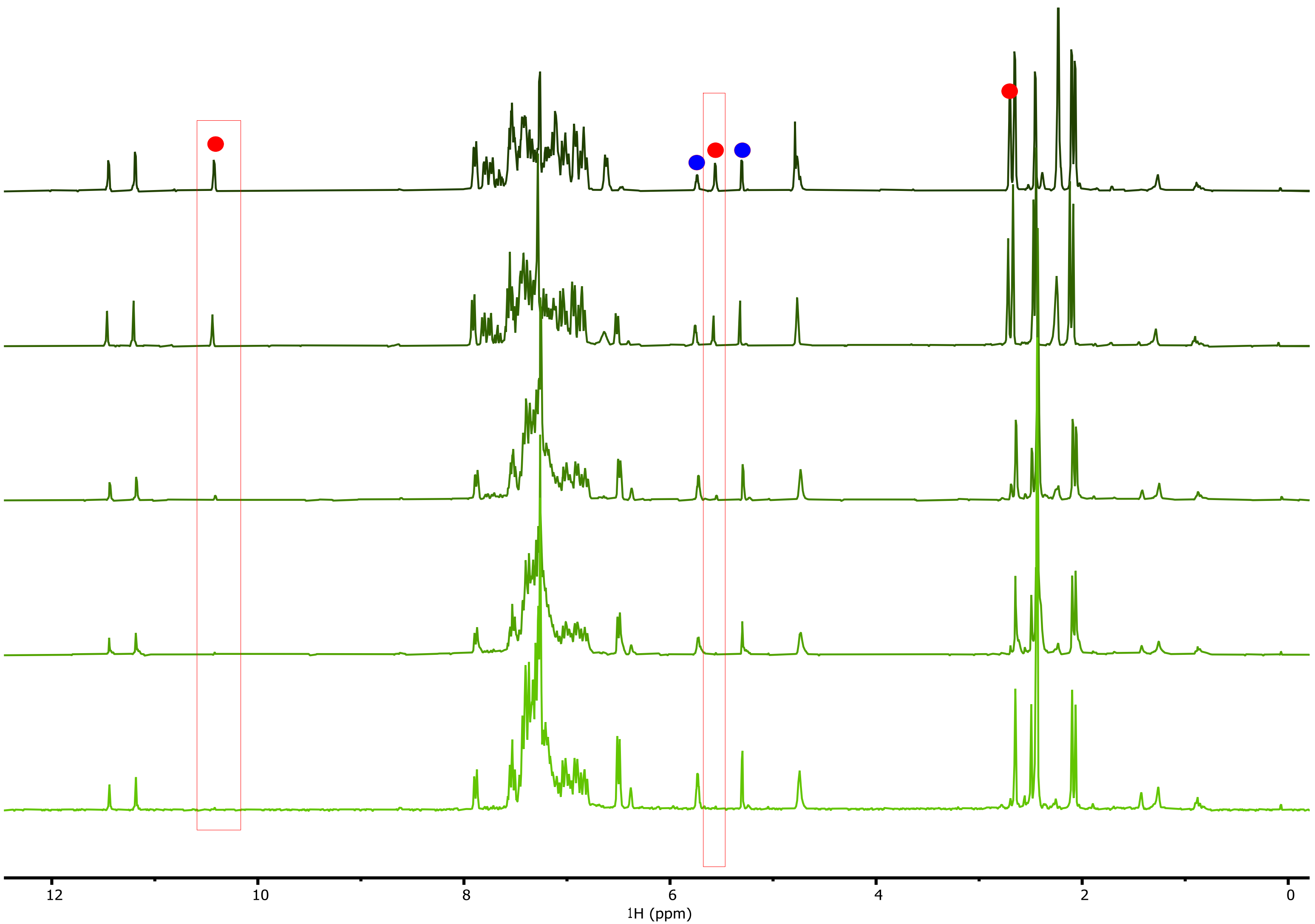


**Figure S5.** $^{1}H$ NMR spectra of the reaction of complex **3** with 2.0 equiv of NaOD in $CDCl_3/D_2O$ mixture after 0.5 h, 3 h, 6 h, 19 h, and 30 h from top to bottom. The disappearance of the Int**1** peaks (red dots) and increase of the $d_2$-styrene peaks (blue dots) can be noticed over the reaction time.

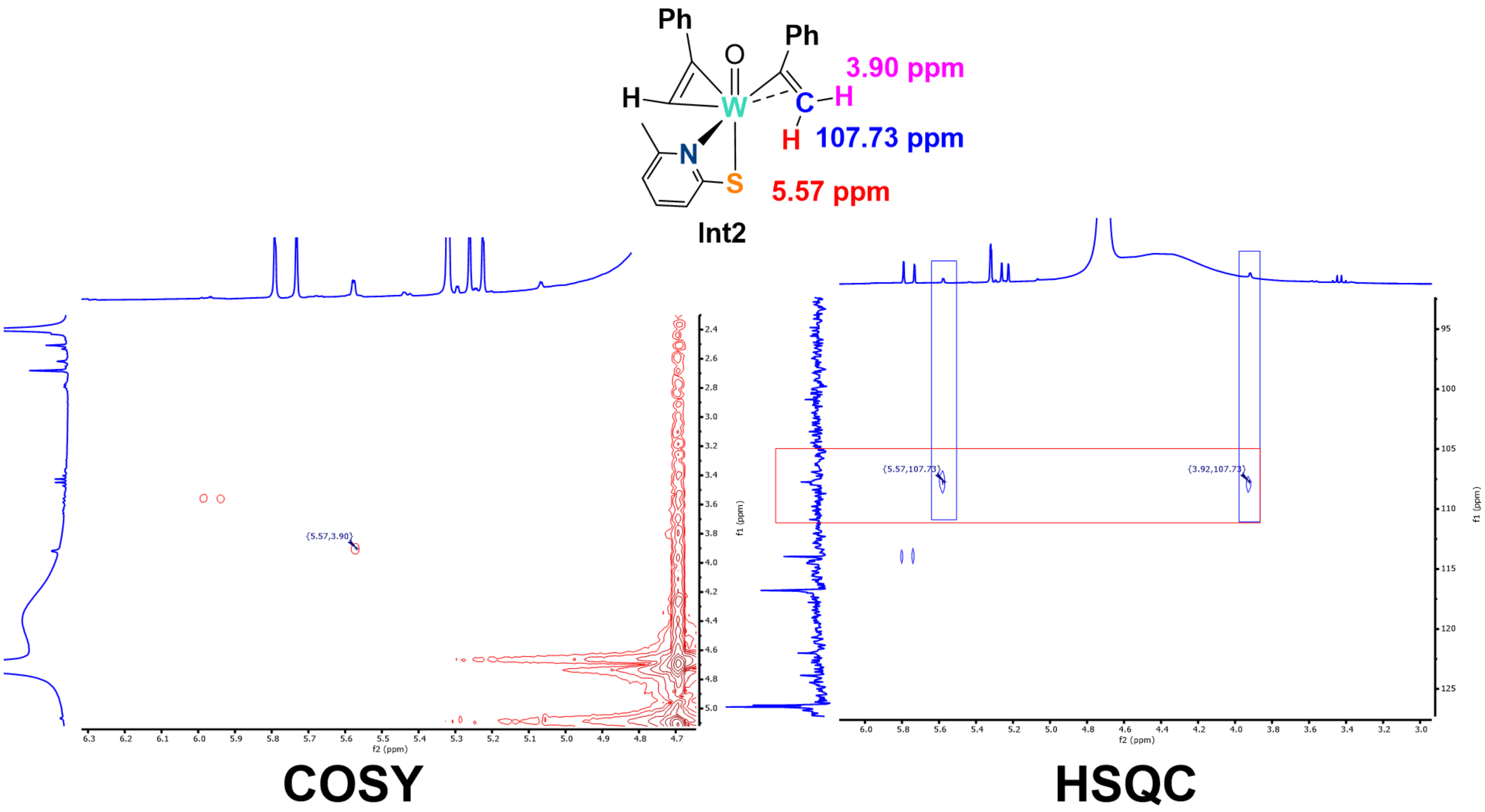


**Figure S6.** 2D spectra (COSY and HSQC) of the reaction of complex 3 with 2.0 equiv of NaOH in $CDCl_3$/H2O mixture after 1 h. COSY shows crosspeak for geminal protons coupling in **Int1**. HSQC shows crosspeaks for both geminal protons with the terminal carbon atom of the vinyl moiety in **Int1**.

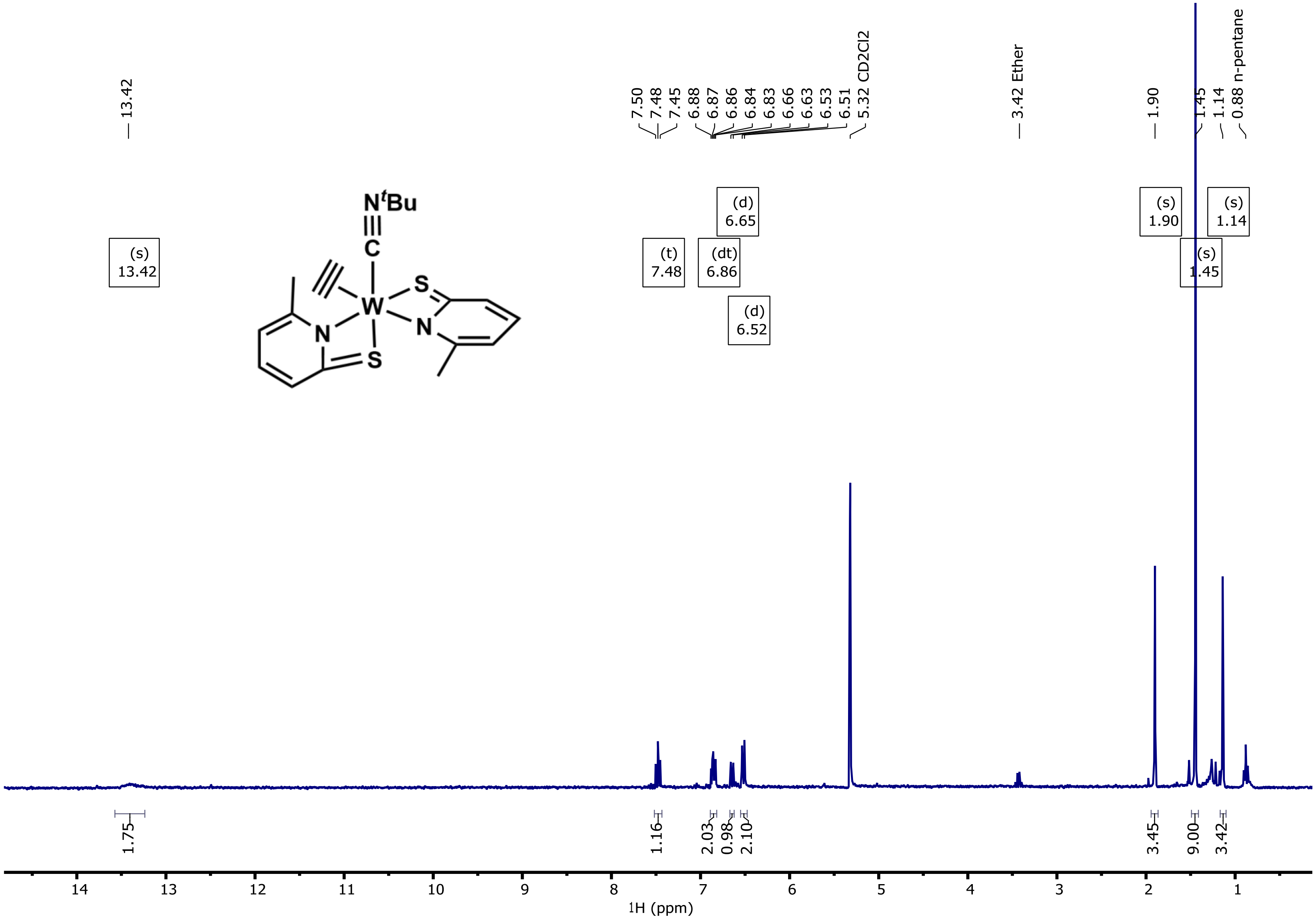


**Figure S7.** $^{1}$H NMR spectrum of [W(CN$^{t}$Bu)($C_2H_2$)(6-MePyS)$_2$] (**4**) in $CD_2Cl_2$. Acetylenic protons exhibit only low resolution at rt.

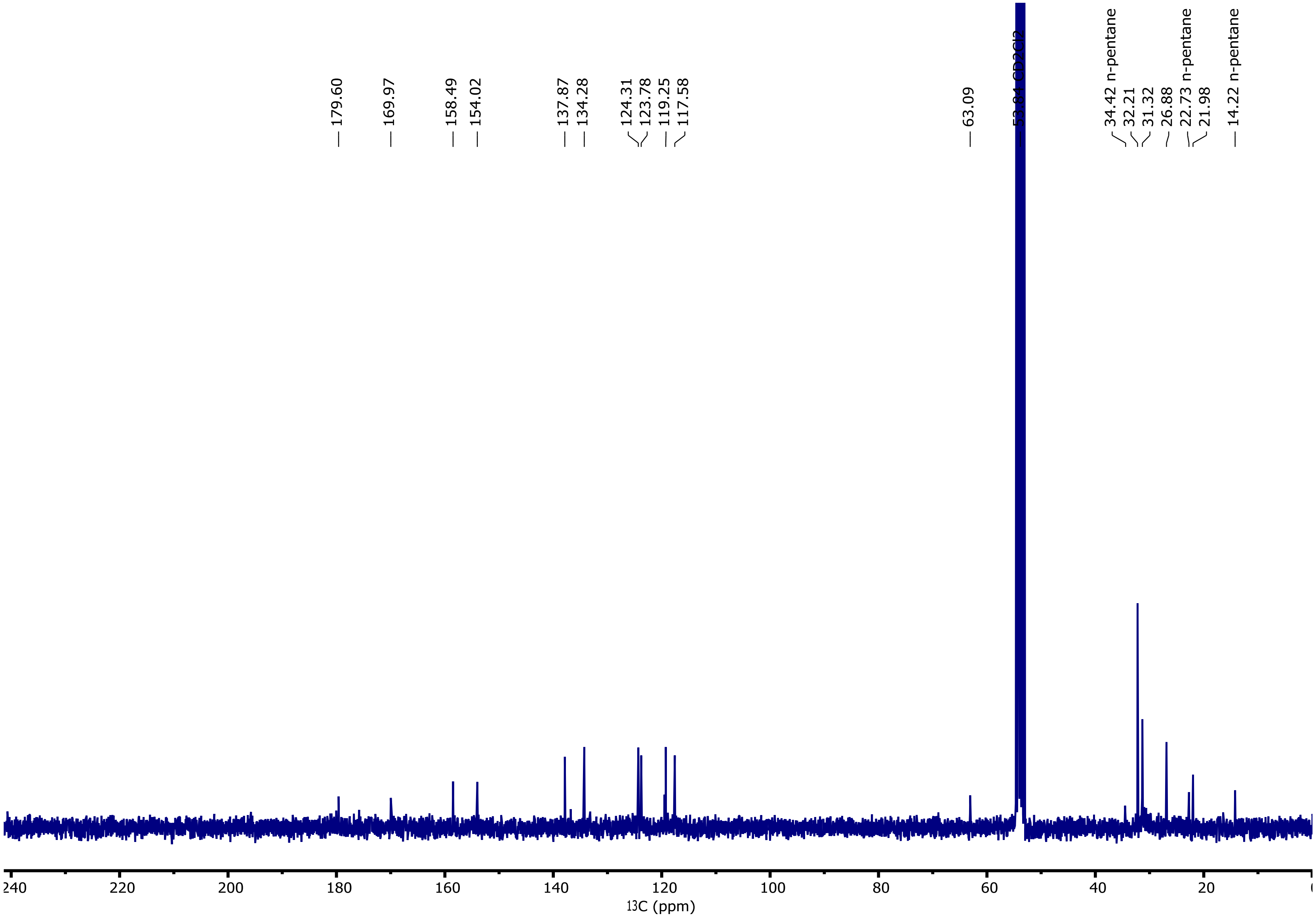


**Figure S8.** $^{13}C$ NMR spectrum of $[W(CN^{t}Bu)(C_2H_2)(6\text{-}MePyS)_2]$ (**4**) in $CD_2Cl_2$. Acetylenic carbons are obscured.

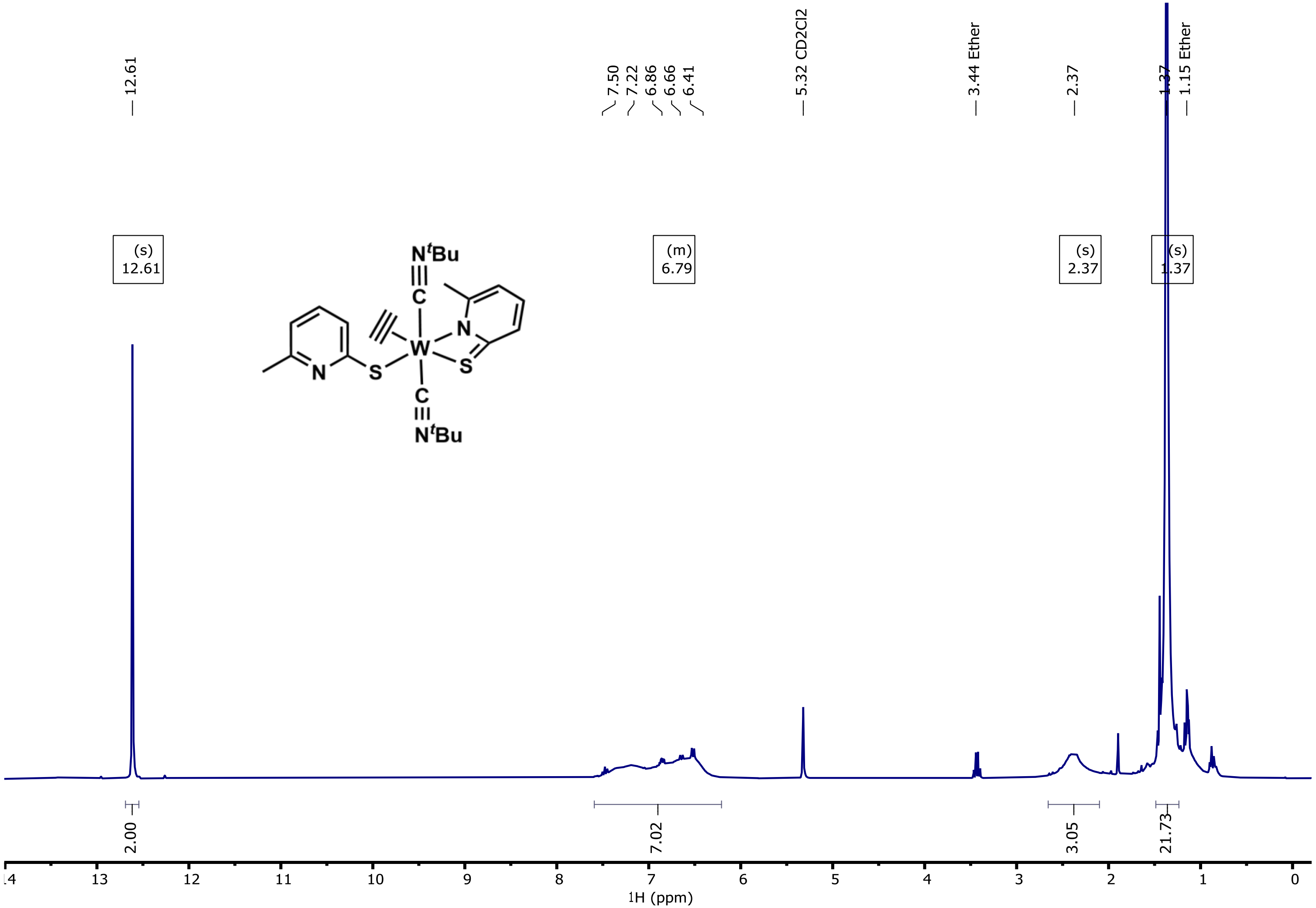


**Figure S9.** $^{1}$H NMR spectrum of [W(CN$^t$Bu)$_2$(C$_2$H$_2$)(6-MePyS)$_2$] (**5**) in $CD_2Cl_2$. Observed dynamic behavior of the 6-MePyS ligands lead to broadened peaks.

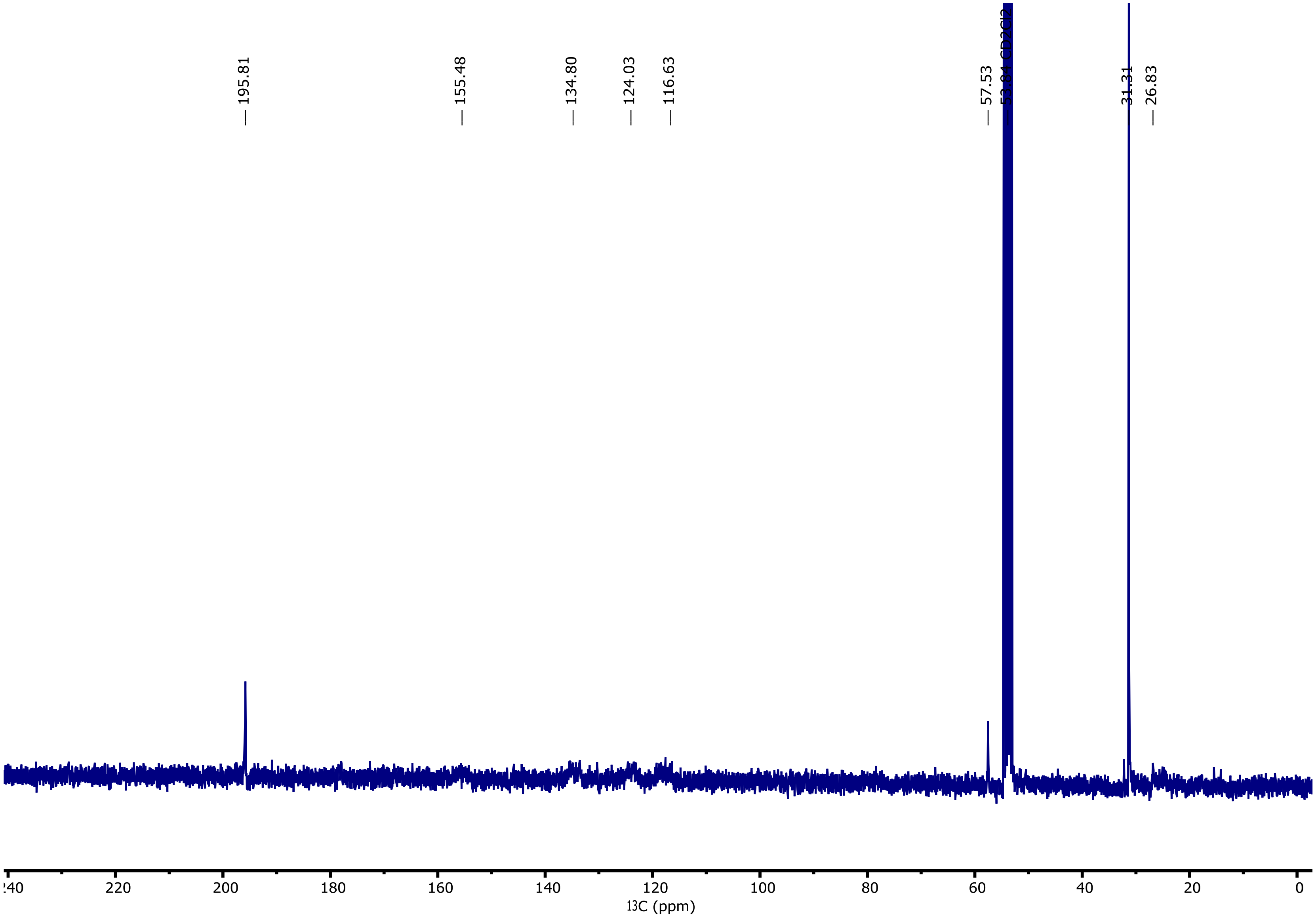


**Figure S10.** $^{13}C$ NMR of [W(CN$^t$Bu)$_2$($C_2H_2$)(6-MePyS)$_2$] (**5**) in $CD_2Cl_2$. The 6-MePyS ligands' carbons are obscured due to the dynamic behavior.

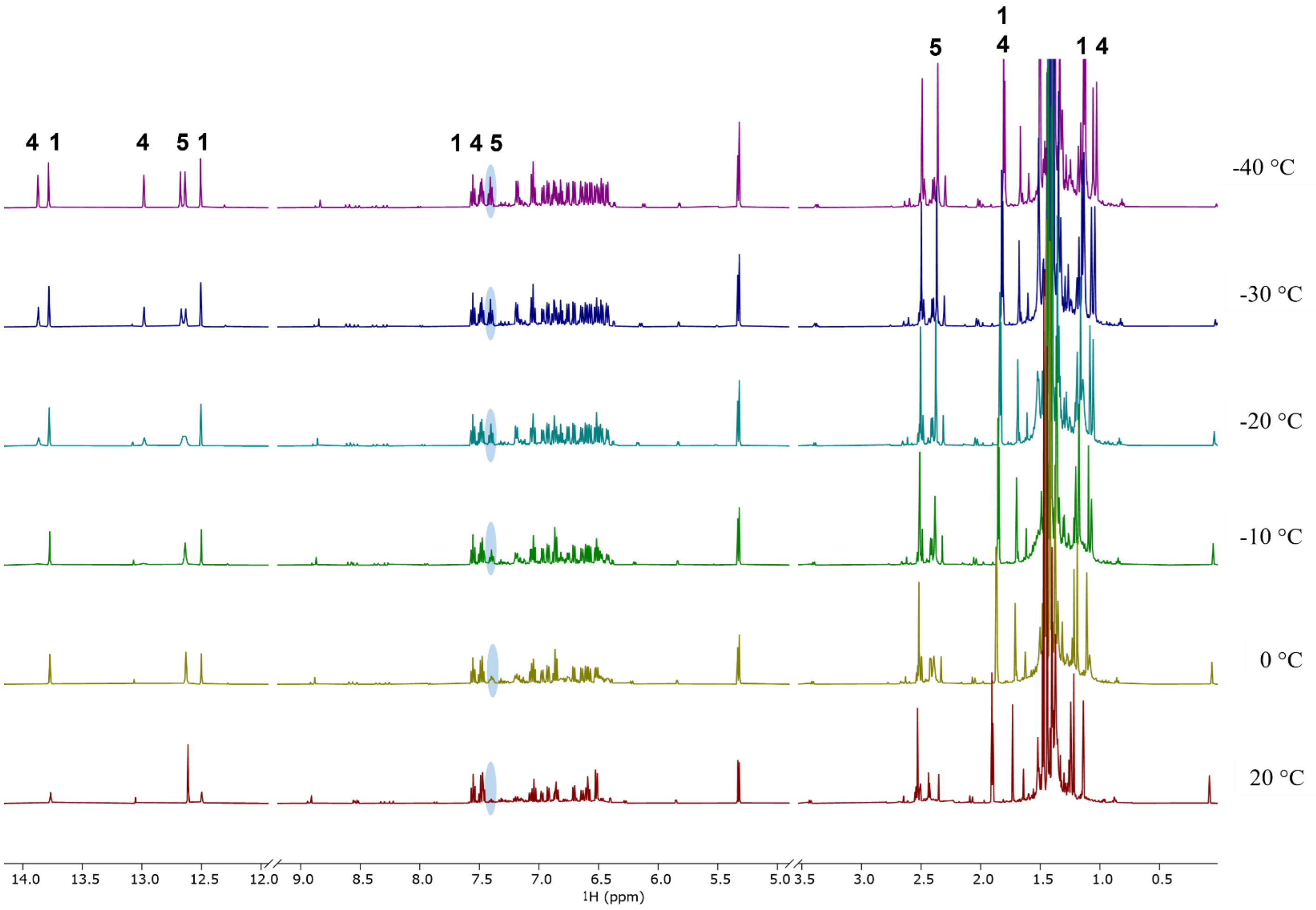


**Figure S11.** Stacked VT $^1$H NMR spectra of the starting complex [W(CO)($C_2H_2$)(6-MePyS)$_2$](**1**), the mono-isocyanide complex [W(CN$^t$Bu)($C_2H_2$)(6-MePyS)$_2$] (**4**) and the dynamic bis-isocyanide complex [W(CN$^t$Bu)$_2$($C_2H_2$)(6-MePyS)(*S*–6-MePyS)] (**5**). Peaks between 14 ppm and 12.5 ppm show the acetylene ligands, the triplets at 7.5 ppm show the *p*-protons of the 6-MePyS ligands and in the aliphatic region the methyl groups of the 6-MePyS ligands of the corresponding complexes are assigned in $CD_2Cl_2$. The integral ration between species **4** and **5** does not change upon varying the temperature.

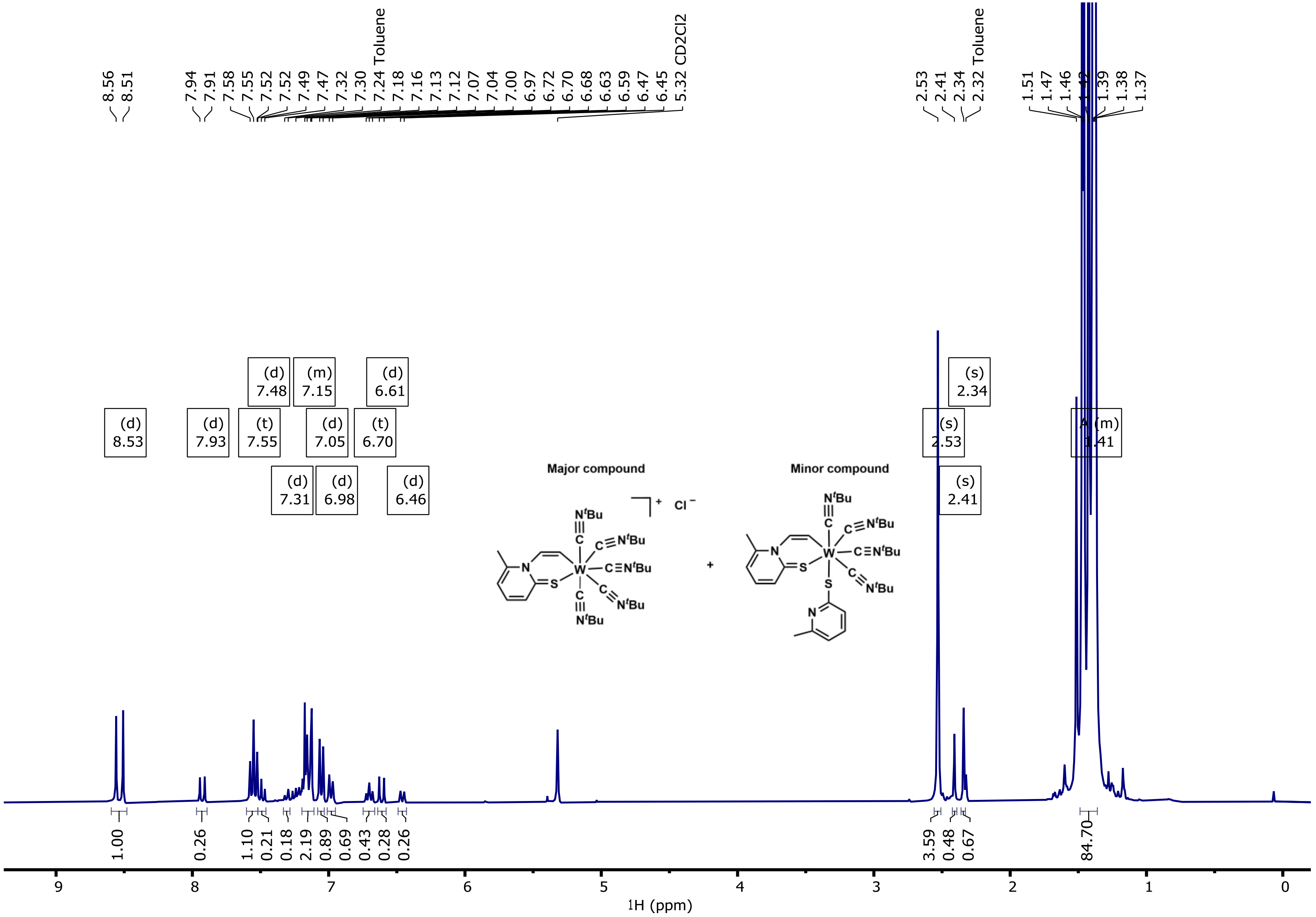


**Figure S12.** $^{1}$H NMR spectrum of [W(*C*,*S*−CHCH−*N*−6-MePyS)(CN$^{t}$Bu)$_5$][Cl] (**6a**) + [W(*C*,*S*−CHCH−*N*−6-MePyS)(*S*−6-MePyS)(CN$^{t}$Bu)$_4$] (**6b**) in $CD_2Cl_2$.

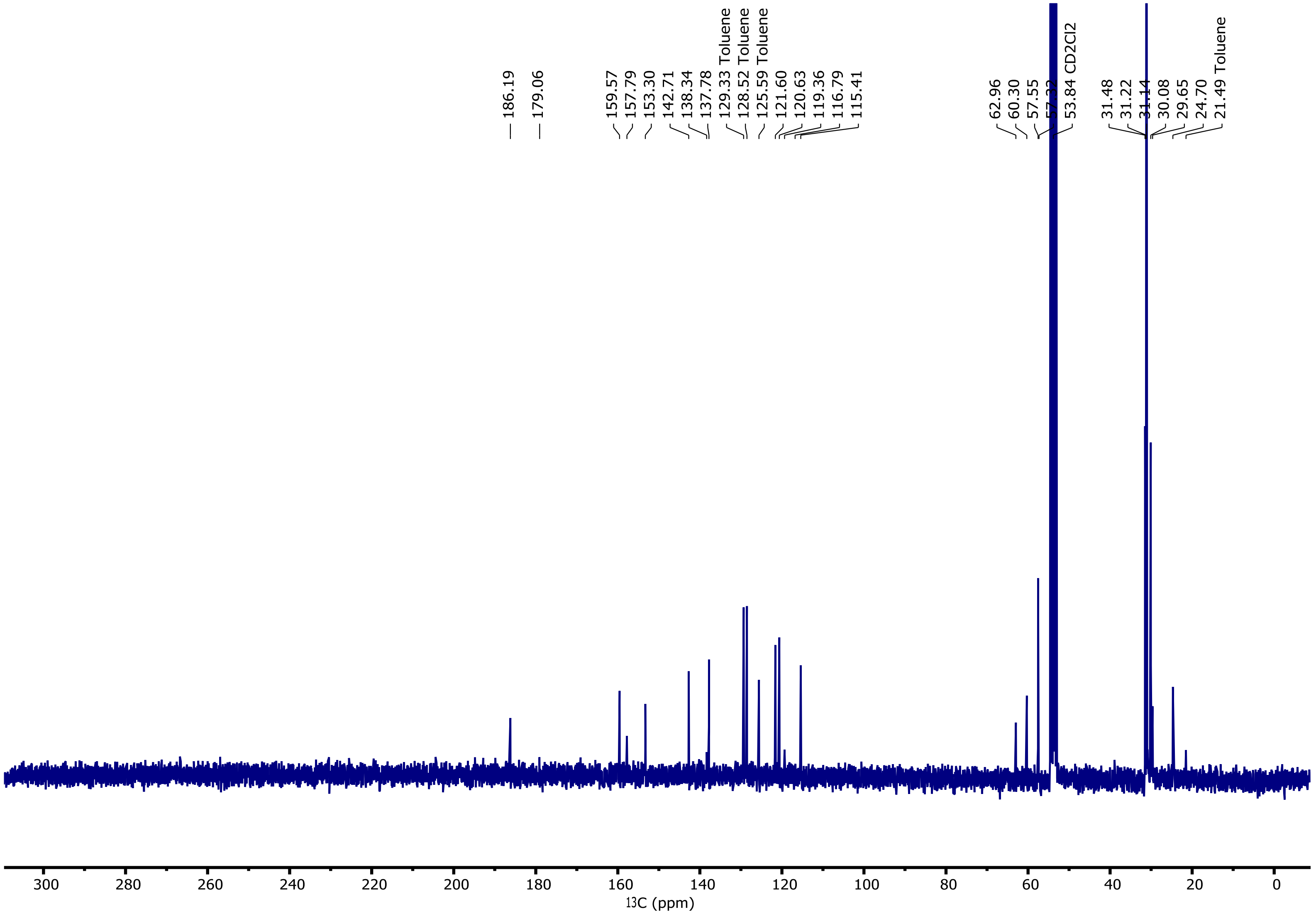


**Figure S13.** $^{13}C$ NMR spectrum of [W(*C*,*S*−CHCH−*N*−6-MePyS)(CN$^t$Bu)$_5$][Cl] (**6a**) + [W(*C*,*S*−CHCH−*N*−6-MePyS)(*S*−6-MePyS)(CN$^t$Bu)$_4$] (**6b**) in $CD_2Cl_2$

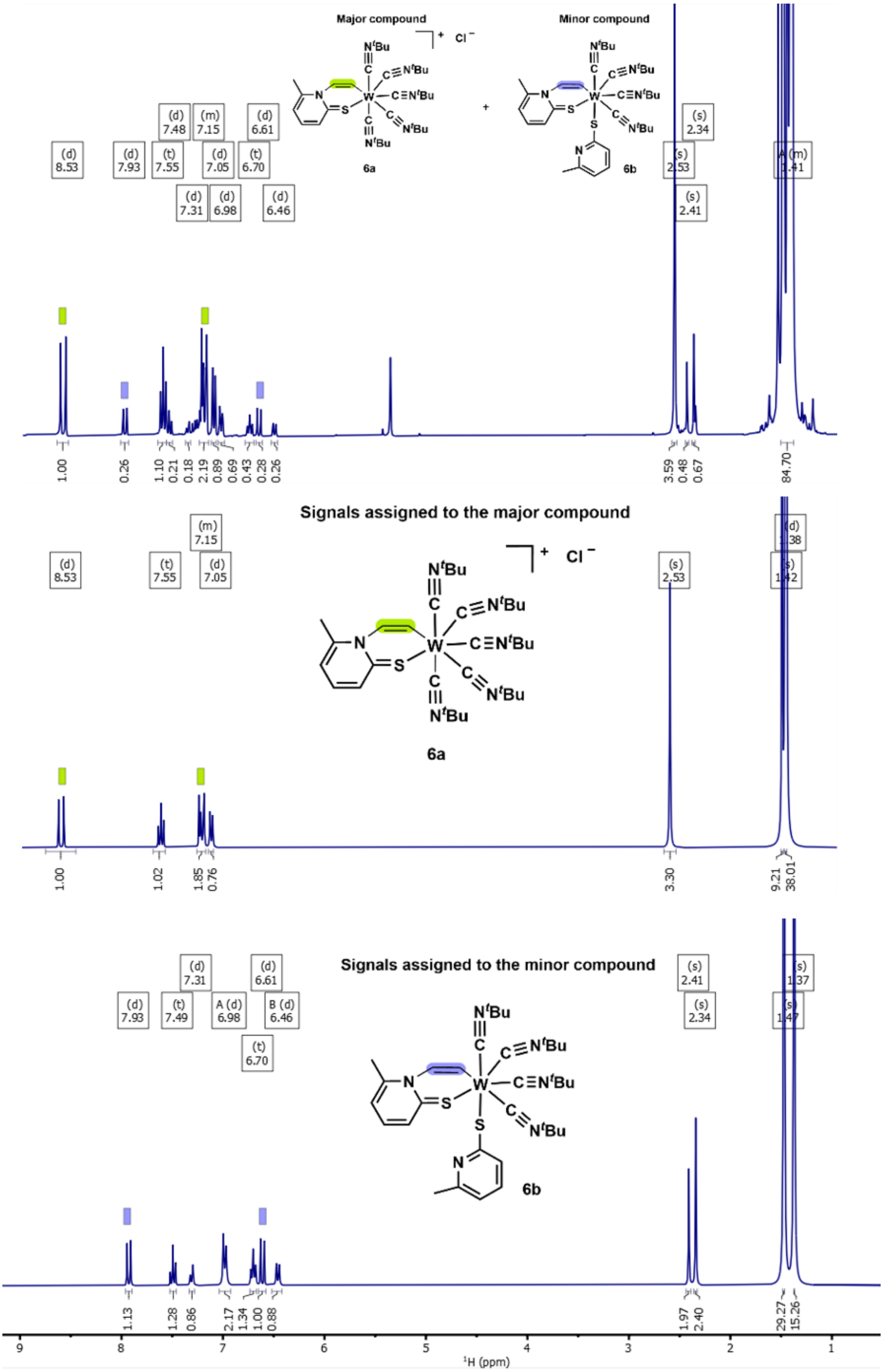


**Figure S14.** Stacked $^1$H NMR spectra of [W(*C*,*S*–CHCH–*N*–6-MePyS)(CN$^t$Bu)$_5$][Cl] (**6a**) + [W(*C*,*S*–CHCH–*N*–6-MePyS)(*S*–6-MePyS)(CN$^t$Bu)$_4$] (**6b**) in $CD_2Cl_2$ with extracted spectra of **6a** and **6b**, respectively.

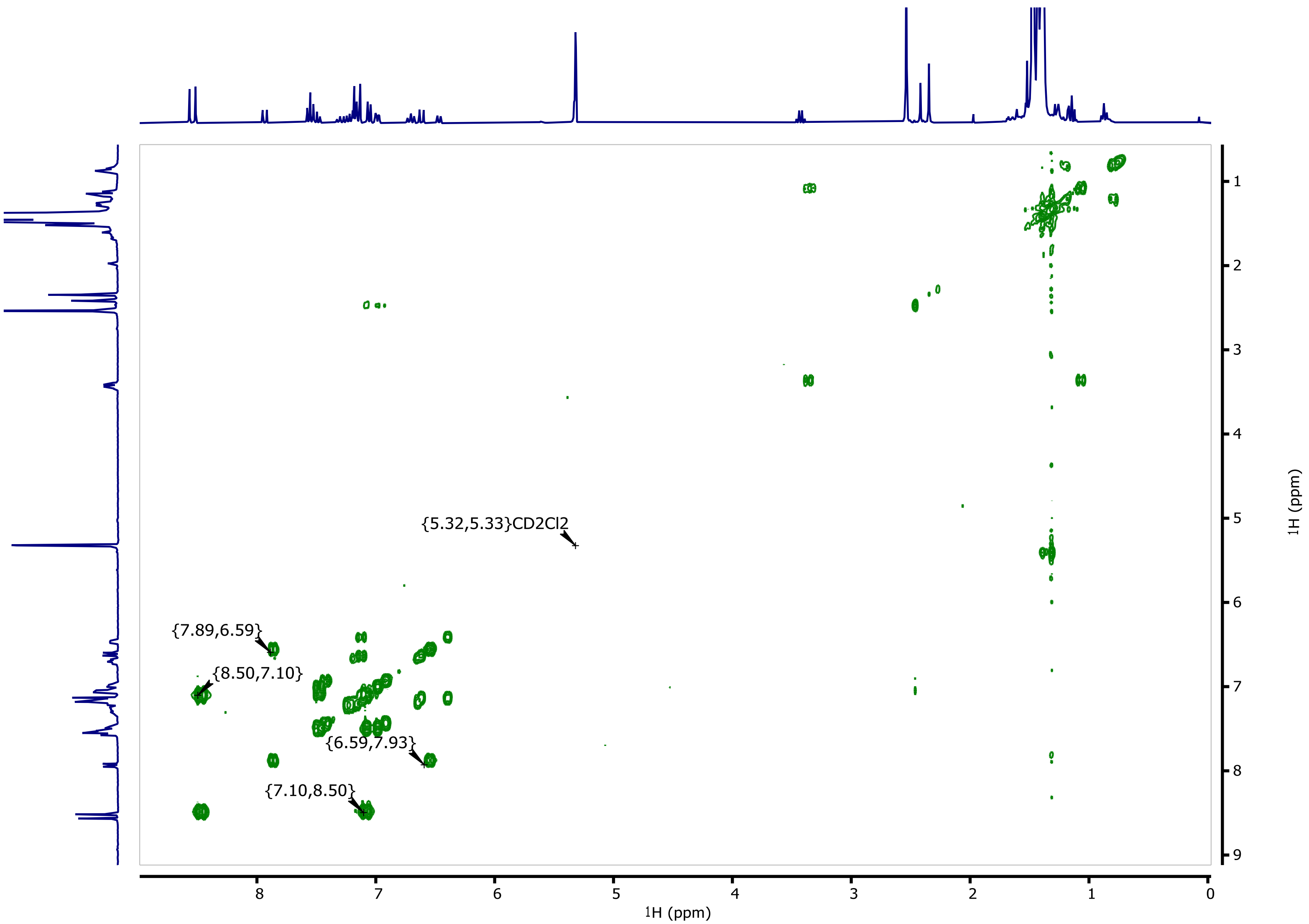


**Figure S15.** $^{1}$H-$^{1}$H COSY spectrum of [W(*C*,*S*−CHCH−*N*−6-MePyS)(CN$^t$Bu)$_5$][Cl] (**6a**) + [W(*C*,*S*−CHCH−*N*−6-MePyS)(*S*−6-MePyS)(CN$^t$Bu)$_4$] (**6b**) in $CD_2Cl_2$.

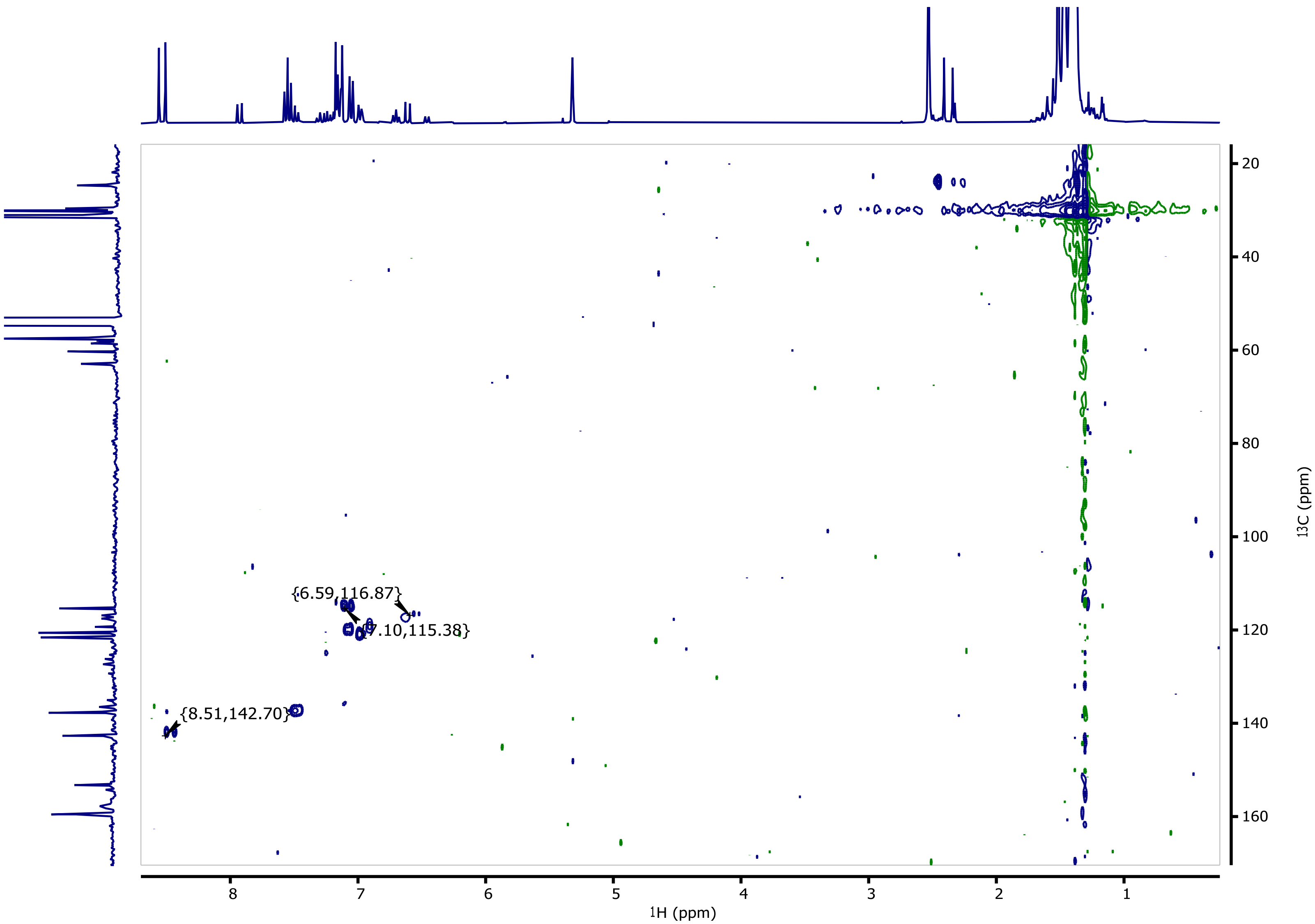


**Figure S16**. $^{1}$H-$^{13}$C HSQC spectrum of [W(*C*,*S*−CHCH−*N*−6-MePyS)(CN$^{t}$Bu)$_5$][Cl] (**6a**) + [W(*C*,*S*−CHCH−*N*−6-MePyS)(*S*−6-MePyS)(CN$^{t}$Bu)$_4$] (**6b**) in $CD_2Cl_2$.

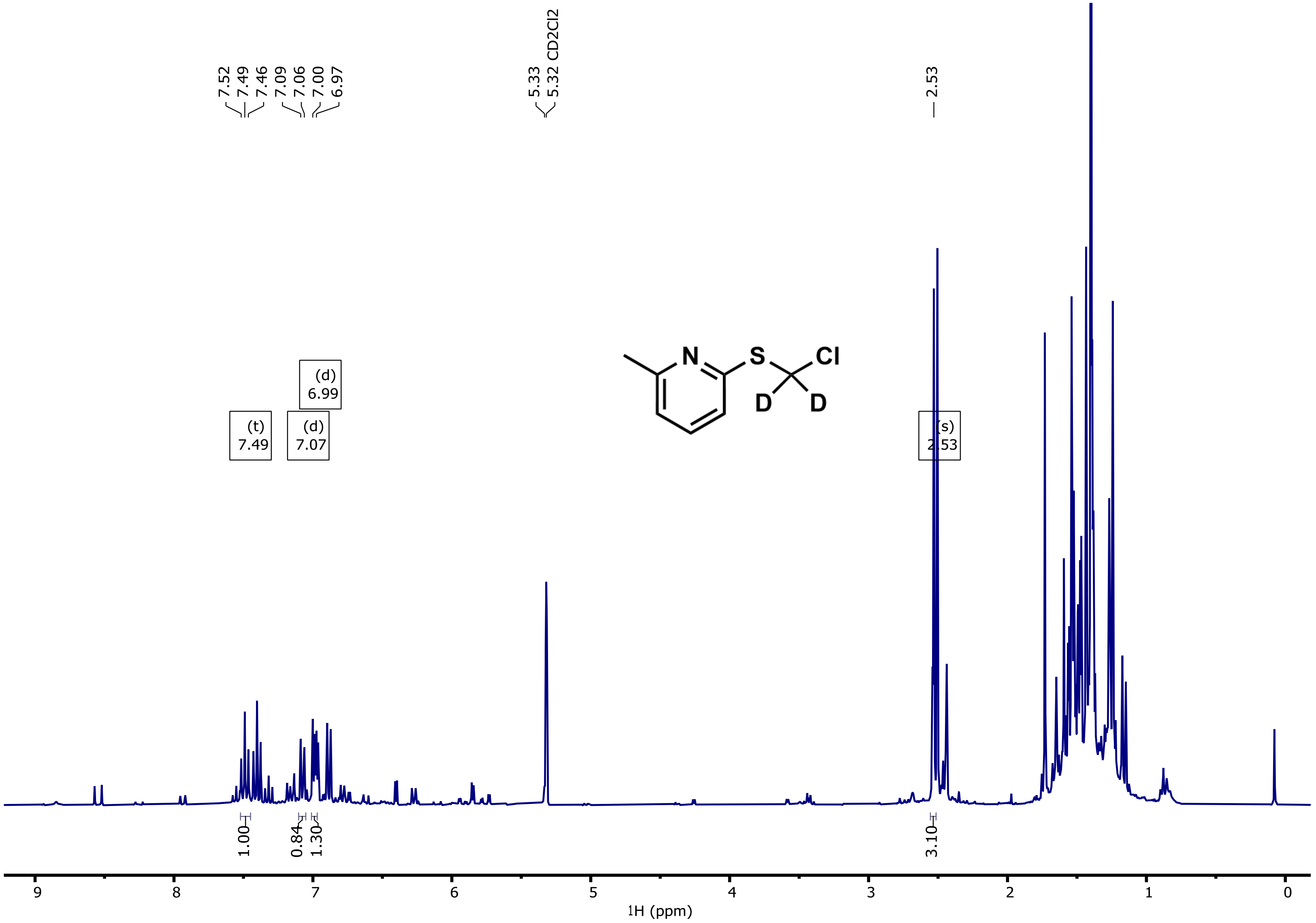


**Figure S17.** Crude reaction mixture of $W(CO)(C_2H_2)(6\text{-}MePyS)_2$ (**1**) and excess of $^t$BuNC in $CD_2Cl_2$ generates side product 6-MePySCD$_2$Cl.

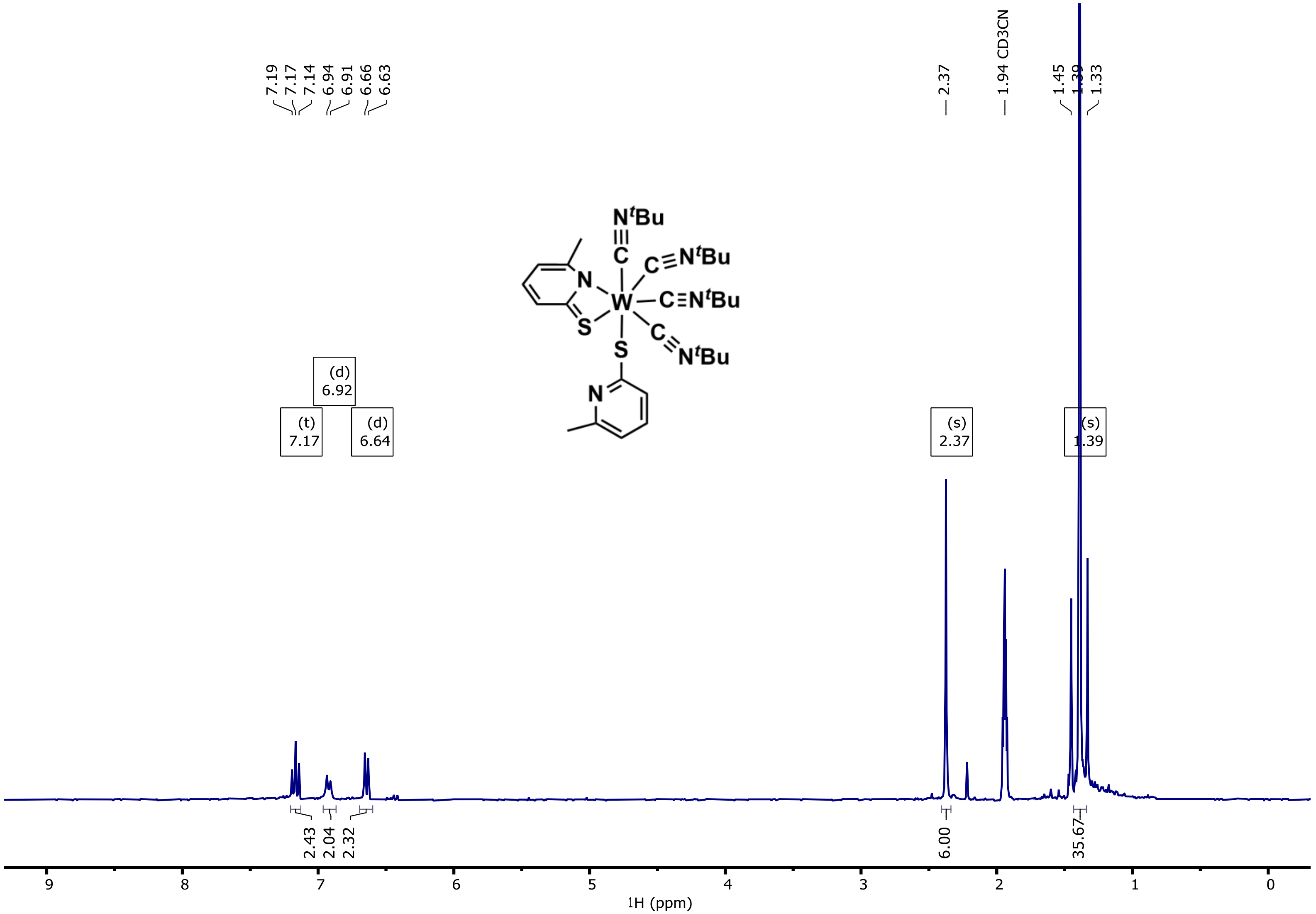


**Figure S18.** $^{1}$H NMR spectrum of [W(CN$^t$Bu)$_4$(6-MePyS)(*S*–6-MePyS)] (**6c**) in $CD_3CN$. The singlets accompanying the $^t$Bu peaks are unidentified impurities of isocyanide.

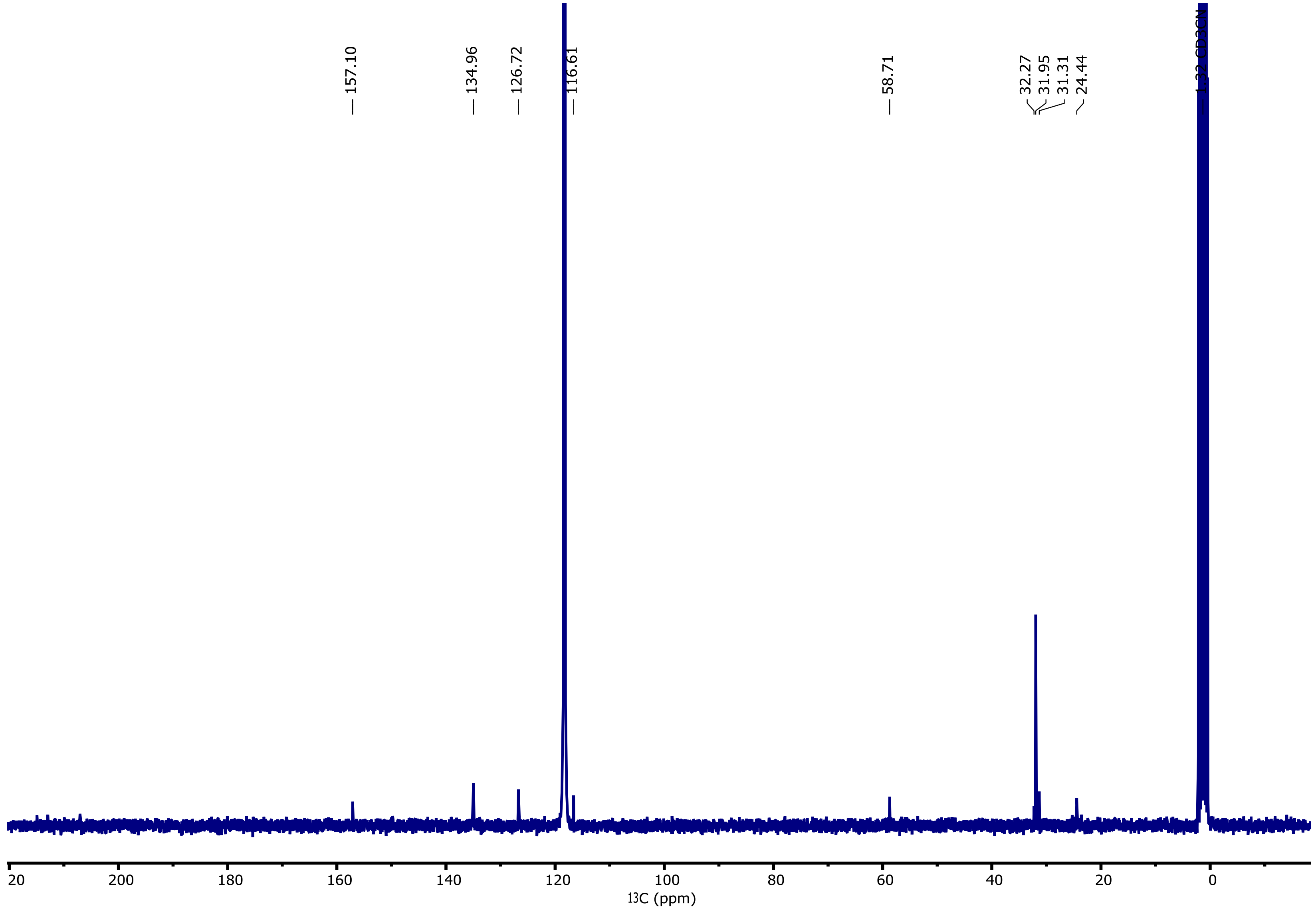


**Figure S19.** $^{13}$C NMR spectrum of [W(CN$^t$Bu)$_4$(6-MePyS)(S−6-MePyS)] (**6c**) in $CD_3CN$.

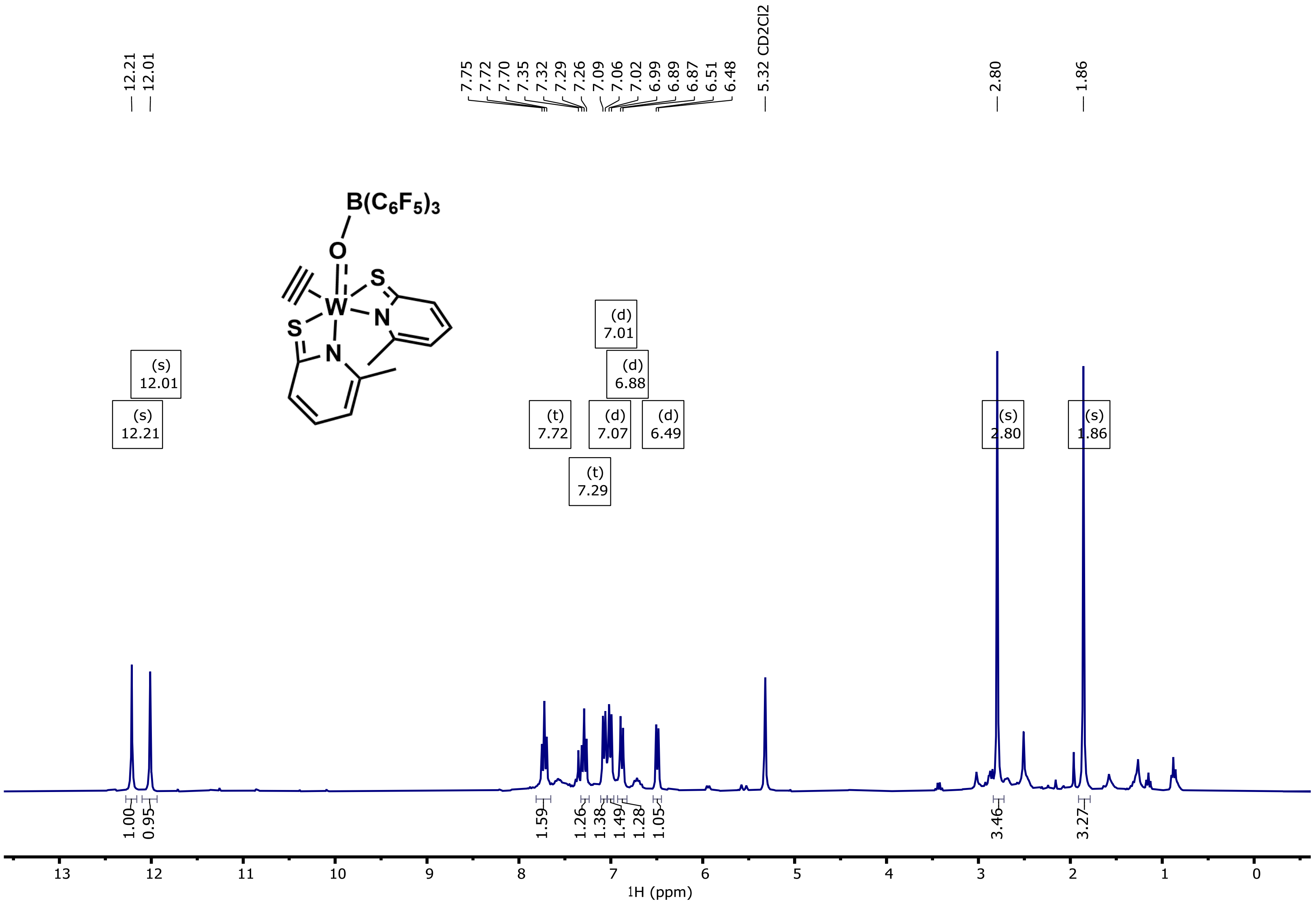


**Figure S20.** $^1$H NMR spectrum of [W{O(B($C_6F_5$)$_3$)}($C_2H_2$)(6-MePyS)$_2$] (**7**) in $CD_2Cl_2$.

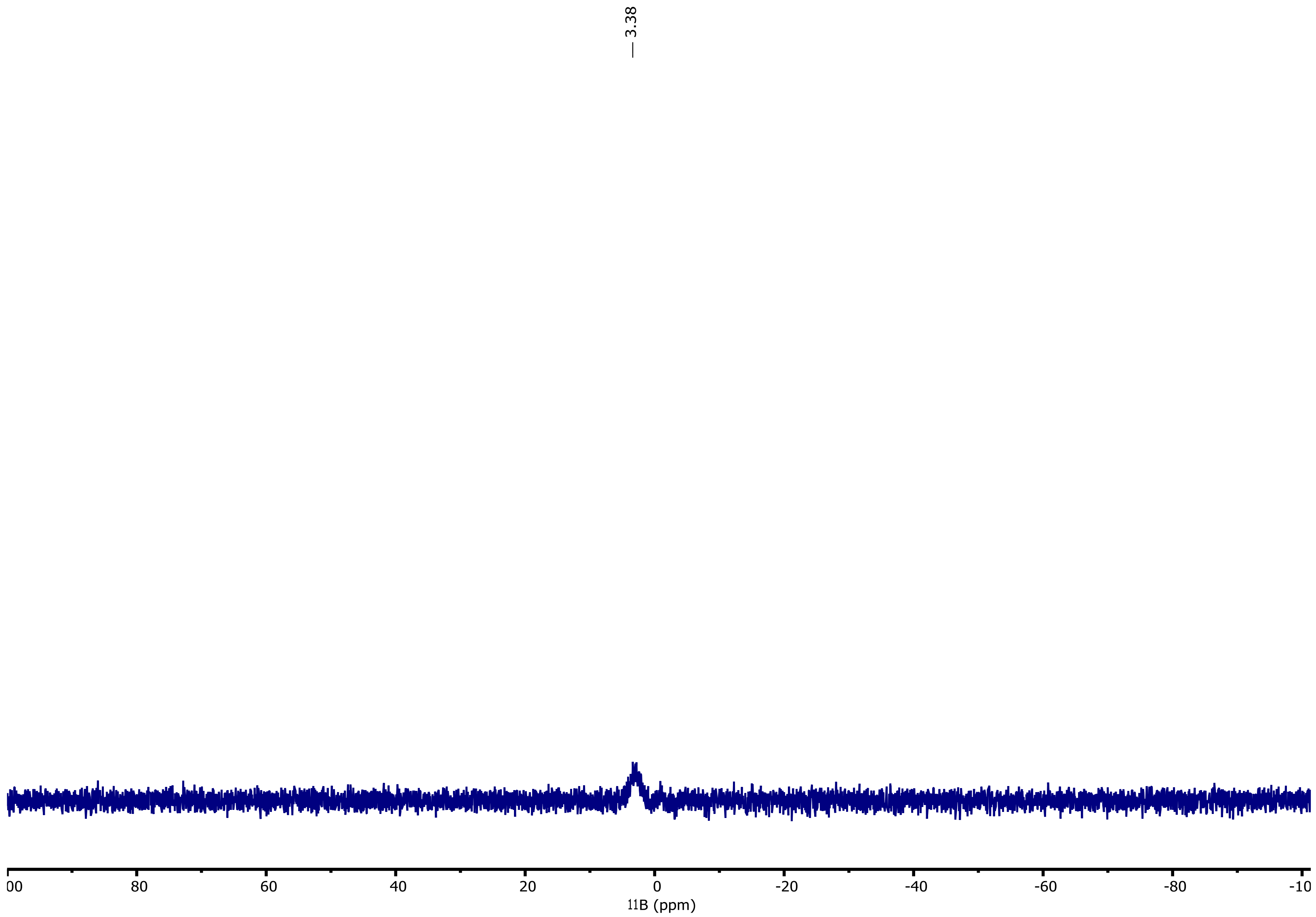


**Figure S21.** $^{11}B$ NMR spectrum [W{O(B($C_6F_5$)$_3$)}($C_2H_2$)(6-MePyS)$_2$] (**7**) in $CD_2Cl_2$.

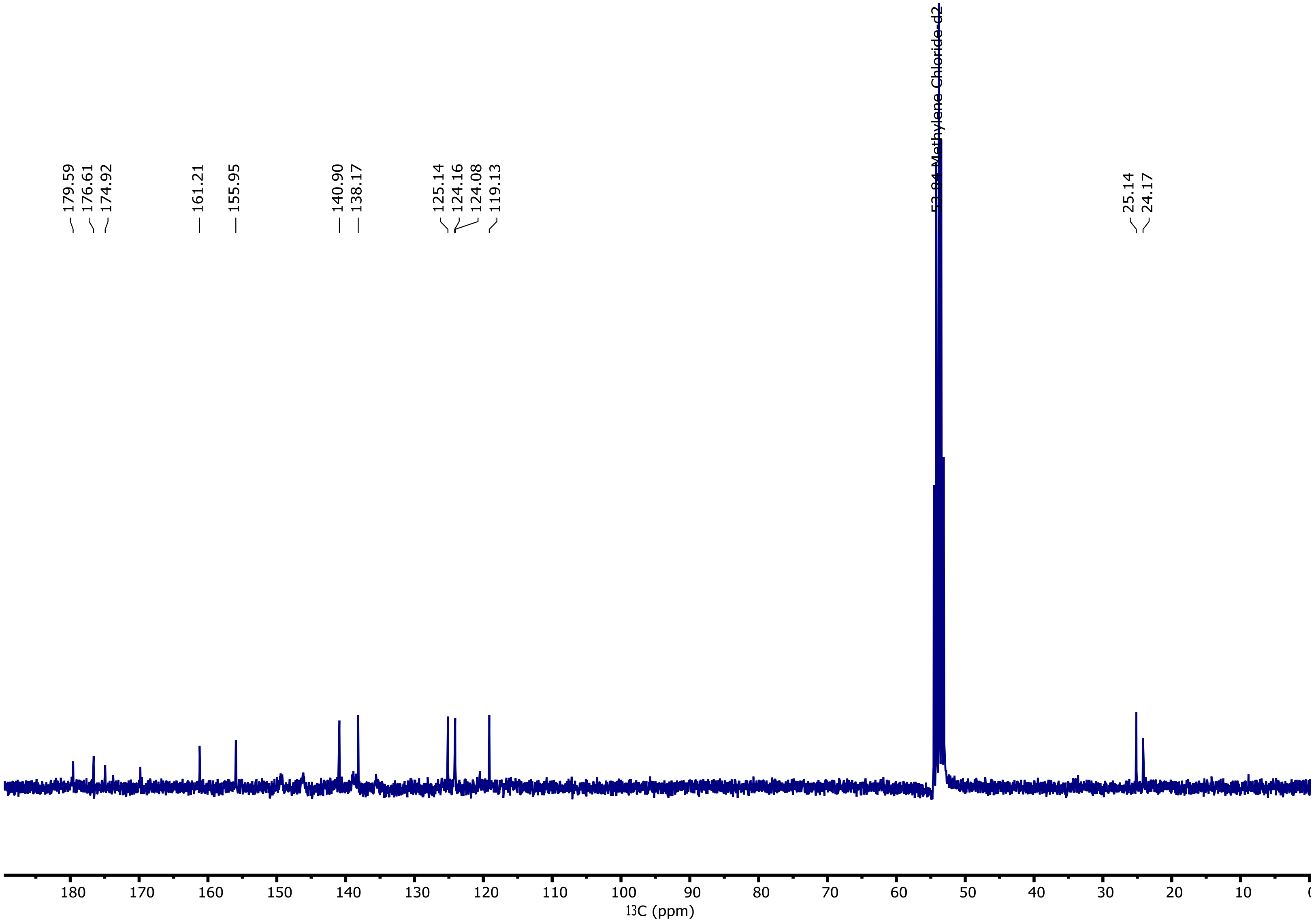


**Figure S22.** $^{13}C$ NMR spectrum of $[W\{O(B(C_6F_5)_3)\}(C_2H_2)(6\text{-MePyS})_2]$ (**7**) in $CD_2Cl_2$.

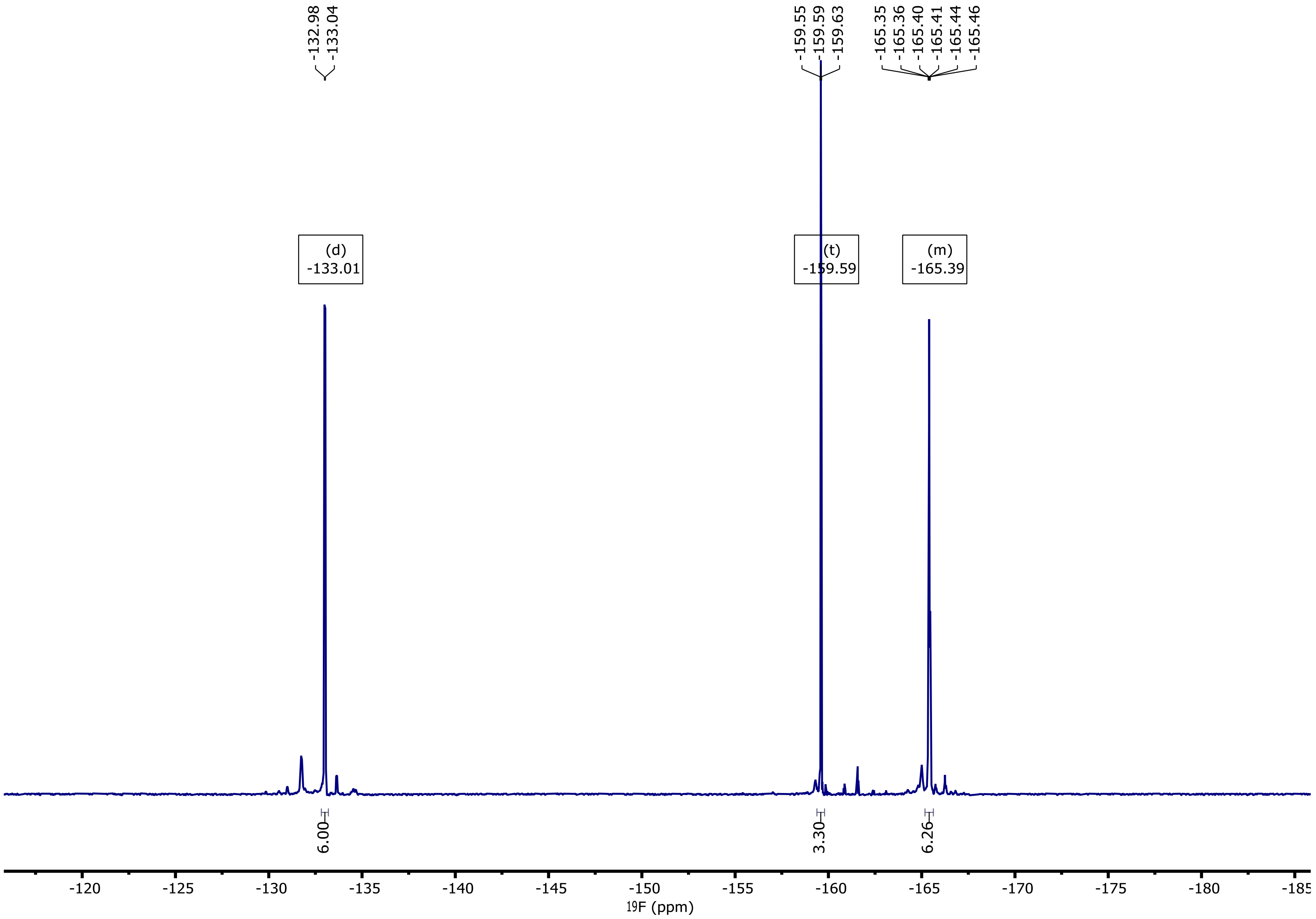


**Figure S23.** $^{19}F$ NMR spectrum of [W{O(B($C_6F_5$)$_3$)}($C_2H_2$)(6-MePyS)$_2$] (**7**) in $CD_2Cl_2$.

# 5 IR Spectrum

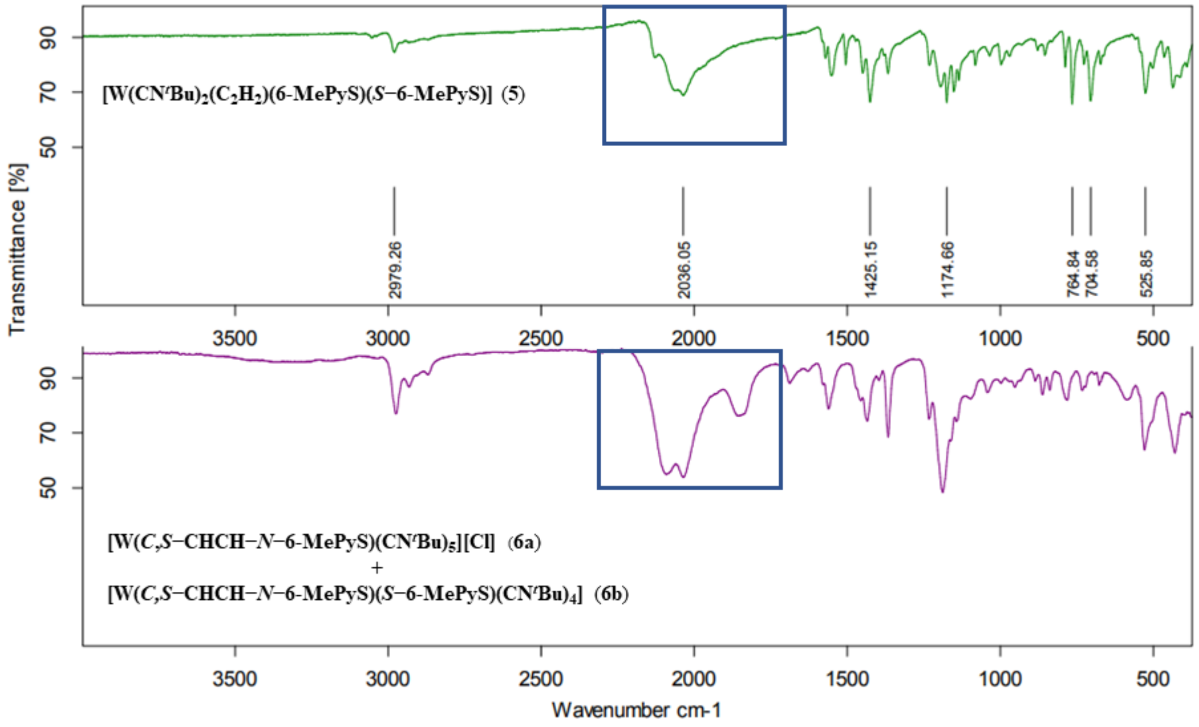


**Figure S24.** Comparison of IR spectra of bis-isocyanide complex **5** (top, green) and acetylene-inserted species **6a**+**6b** (bottom, purple).

# 6 Line Shape Analysis

The line shape analysis allowed extraction of thermodynamic parameters for the acetylene rotation in complex **4**. The data was collected as described in literature.[7] By using the Eyring equation, ΔH (slope = -ΔH/R) and ΔS (intercept = $\ln(k_b/h)$+ΔS/R) were determined. Subsequently, ΔG (ΔH-TΔS) for the acetylene rotation in **4** at 298 K was calculated to be 13.4 kcal/mol.

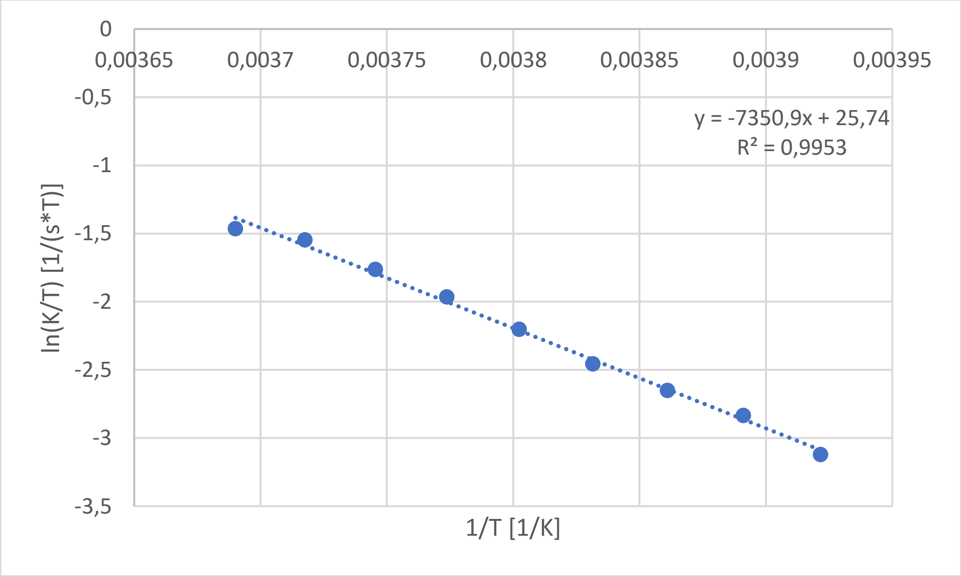


**Figure S25.** Line shape analysis for compound **4** based on the Eyring equation.

# 7 Crystal Structure Determination

**General.** X-ray structural analysis was performed using monochromatized MoKα radiation at 100 K on a Bruker APEX-II CCD (**5** and **7**) and GaKα radiation on a Bruker D8 Venture diffractometer (**6c**). Bruker APEX2 software[8] was used to collect and reduce data and determine the space group. Absorption corrections were applied using SADABS.[9] The structure was solved with the SHELXT (**5** and **7**)[10] and SHELXS (**6c**)[11] and refined by full-matrix least-squares techniques against $F^2$ (SHELXL 2019/2)[12] using the Olex2 software.[13]

**Table S4.** Crystal data and structure refinement for [W(CN$^t$Bu)$_2$($C_2H_2$)(6-MePyS)(*S*–6-MePyS)] (**5**), [W(CN$^t$Bu)$_4$(6-MePyS)(*S*–6-MePyS)] (**6c**) and [W{O(B($C_6F_5$)$_3$)}($C_2H_2$)(6-MePyS)$_2$] (**7**)

| **Compound** | **5** | **6c** | **7** |
|---|---|---|---|
| **CCDC n°** | 2320791 | 2320792 | 2320790 |
| **Empirical formula** | $C_{24}H_{32}N_4S_2W$ | $C_{32}H_{48}N_6S_2W$ | $C_{32}H_{14}BF_{15}N_2OS_2W$ |
| **Formula weight** | 624.50 | 764.73 | 986.23 |
| **Temperature /K** | 100.16 | 101.00 | 100.03 |
| **Crystal system** | monoclinic | monoclinic | monoclinic |
| **Space group** | $P2_1/c$ | Cc | $P2_1/c$ |
| **a /Å** | 15.4792(8) | 20.6064(8) | 10.9366(5) |
| **b /Å** | 10.1971(6) | 10.1799(4) | 16.4237(7) |
| **c /Å** | 16.8084(10) | 17.5772(7) | 18.2315(9) |
| **α /°** | 90 | 90 | 90 |
| **β /°** | 104.719(2) | 107.9500(10) | 94.745(2) |
| **γ /°** | 90 | 90 | 90 |
| **Volume /Å$^3$** | 2566.0(3) | 3507.7(2) | 3263.5(3) |
| **Z** | 4 | 4 | 4 |
| **$\rho_{calc}$ g/cm$^3$** | 1.617 | 1.448 | 2.007 |
| **μ /mm$^{-1}$** | 4.682 | 5.059 | 3.786 |
| **F(000)** | 1240.0 | 1552.0 | 1896.0 |
| **Crystal size /mm$^3$** | 0.27 × 0.25 × 0.15 | 0.37× 0.35 × 0.24 | 0.34 × 0.25 × 0.15 |

| | | | |
|---|---|---|---|
| **Radiation** | MoKα ($\lambda$ = 0.71073) | GaKα ($\lambda$ = 1.34139) | MoKα ($\lambda$ = 0.71073) |
| **2Θ range for data collection/°** | 4.716 to 57.4 | 8.514 to 126.818 | 4.484 to 60.066 |
| **Index ranges** | $-19 \leq h \leq 20$, $-13 \leq k \leq 13$, $-22 \leq l \leq 22$ | $-27 \leq h \leq 27$, $-13 \leq k \leq 13$, $-23 \leq l \leq 22$ | $-15 \leq h \leq 15$, $-22 \leq k \leq 23$, $-25 \leq l \leq 25$ |
| **Reflections collected** | 60342 | 54691 | 80573 |
| **Independent reflections** | 6630 [$R_{int}$ = 0.1196, $R_{sigma}$ = 0.0689] | 8462 [$R_{int}$ = 0.0289, $R_{sigma}$ = 0.0230] | 9548 [$R_{int}$ = 0.0747, $R_{sigma}$ = 0.0427] |
| **Data/restraints/parameters** | 6630/6/304 | 8462/2/385 | 9548/0/489 |
| **Goodness-of-fit on $F^2$** | 1.033 | 1.071 | 1.064 |
| **Final R indexes [I>=2σ (I)]** | $R_1$ = 0.0306, $wR_2$ = 0.0488 | $R_1$ = 0.0133, $wR_2$ = 0.0328 | $R_1$ = 0.0377, $wR_2$ = 0.0823 |
| **Final R indexes [all data]** | $R_1$ = 0.0629, $wR_2$ = 0.0572 | $R_1$ = 0.0133, $wR_2$ = 0.0328 | $R_1$ = 0.0502, $wR_2$ = 0.0888 |
| **Largest diff. peak/hole / e Å$^{-3}$** | 1.77/-1.24 | 0.27/-0.83 | 5.50/-2.71 |

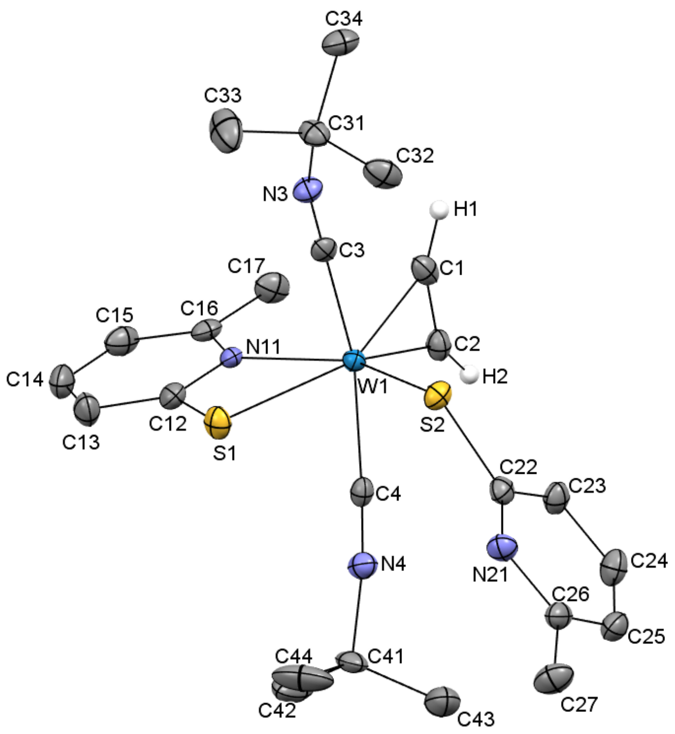


**Figure S26.** Molecular structure of [W(CN$^t$Bu)$_2$(C$_2$H$_2$)(6-MePyS)(*S*–6-MePyS)] (**5**) showing the atomic numbering scheme. The ellipsoids are drawn at the 50% probability level. Except for those of the acetylene group, the H atoms were omitted for clarity.

**Table S5.** Selected bond lengths [Å] for [W(CN$^t$Bu)$_2$(C$_2$H$_2$)(6-MePyS)(*S*–6-MePyS)] (**5**)

| | | | |
|---|---|---|---|
| **W1-C1** | 2.033(4) | **C1-C2** | 1.290(5) |
| **W1-C2** | 2.024(4) | **N3-C3** | 1.165(5) |
| **W1-S1** | 2.6051(10) | **N3-C31** | 1.474(4) |
| **W1-N11** | 2.221(3) | **N4-C4** | 1.159(4) |
| **W1-S2** | 2.3828(10) | **N4-C41** | 1.49(8) |
| **W1-C3** | 2.083(4) | **S1-C12** | 1.729(4) |
| **W1-C4** | 2.111(4) | **S2-C22** | 1.785(4) |

**Table S6.** Selected bond angles [°] for [W(CN$^t$Bu)$_2$(C$_2$H$_2$)(6-MePyS)(*S*–6-MePyS)] (**5**)

| | | | |
|---|---|---|---|
| **C2-W1-C1** | 37.06(15) | **C3-W1-S1** | 83.08(10) |
| **C2-C1-W1** | 71.1(2) | **C3-W1-N11** | 93.68(12) |
| **C1-C2-W1** | 71.8(3) | **C3-W1-S2** | 83.18(10) |
| **C1-W1-C3** | 79.22(15) | **C3-W1-C4** | 161.15(14) |

| | | | |
|---|---|---|---|
| **C1-W1-C4** | 119.43(16) | S2-W1-S1 | 88.95(3) |
| **C2-W1-C3** | 116.26(15) | **C4-W1-S1** | 79.77(10) |
| **C2-W1-C4** | 82.37(15) | **C4-W1-N11** | 85.74(12) |
| **C1-W1-S1** | 155.83(12) | **C4-W1-S2** | 88.72(10) |
| **C1-W1-N11** | 100.96(13) | **C12-S1-W1** | 80.19(13) |
| **C1-W1-S2** | 105.02(10) | **C12-N11-W1** | 104.0(2) |
| **C2-W1-S1** | 155.84(10) | **C22-S2-W1** | 112.46(12) |
| **C2-W1-N11** | 98.88(13) | **C3-N3-C31** | 155.7(4) |
| **C2-W1-S2** | 106.84(11) | **N3-C3-W1** | 176.8(3) |
| **N11-W1-S1** | 63.77(8) | **C4-N4-C41** | 167.7(11) |
| **N11-W1-S2** | 152.71(8) | **N4-C4-W1** | 174.1(3) |

**Table S7.** Selected torsion angles [°] for [W(CN$^t$Bu)$_2$(C$_2$H$_2$)(6-MePyS)(*S*–6-MePyS)] (**5**)

| | | | |
|---|---|---|---|
| **W1-S1-C12-N11** | -2.7(2) | **W1-N11-C16-C17** | -3.1(5) |
| **W1-S1-C12-C13** | 177.8(4) | **W1-S2-C22-N21** | -135.4(2) |
| **W1-N11-C12-S1** | 3.2(3) | **W1-S2-C22-C23** | 52.0(3) |
| **W1-N11-C12-C13** | -177.2(3) | **C3-N3-C31-C33** | -117.9(9) |
| **W1-N11-C16-C15** | 177.9(3) | **C4-N4-C41-C43** | -125(6) |

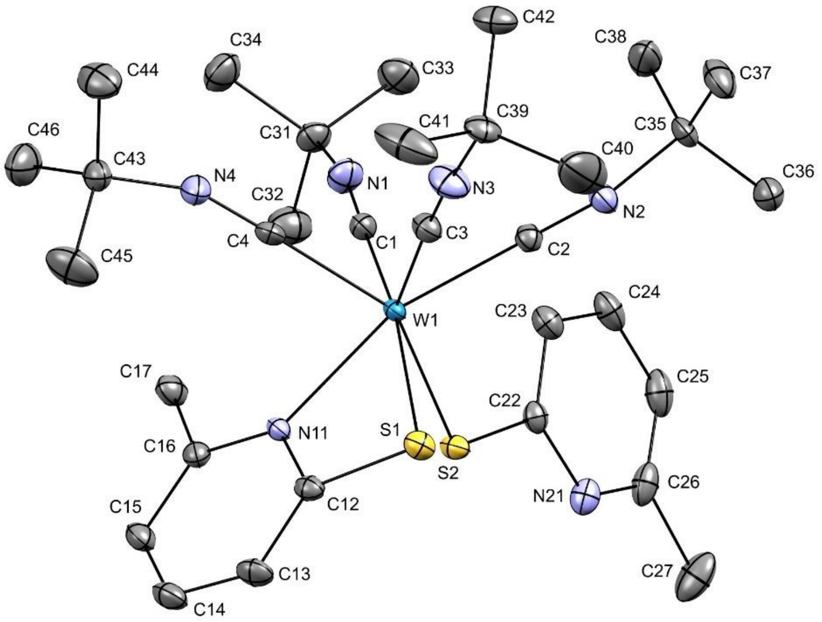


**Figure S27.** Molecular structure of [W(CN$^t$Bu)$_4$(6-MePyS)(*S*–6-MePyS)] (**6c**) showing the atomic numbering scheme. The ellipsoids are drawn at the 50% probability level. The H atoms were omitted for clarity.

**Table S8.** Selected bond lengths [Å] for [W(CN$^t$Bu)$_4$(6-MePyS)(*S*–6-MePyS)] (**6c**)

| | | | |
|---|---|---|---|
| **W1-C1** | 2.037(2) | **C1-N1** | 1.168(3) |
| **W1-C2** | 2.083(2) | **N1-C31** | 1.446(3) |
| **W1-C3** | 2.055(3) | **C2-N2** | 1.157(3) |
| **W1-C4** | 2.027(2) | **N2-C35** | 1.462(3) |
| **W1-S1** | 2.6160(6) | **C3-N3** | 1.163(4) |
| **W1-N11** | 2.2396(17) | **N3-C39** | 1.442(3) |
| **W1-S2** | 2.5082(5) | **C4-N4** | 1.179(3) |
| **S1-C12** | 1.727(2) | **N4-C43** | 1.456(3) |
| **S2-C22** | 1.762(2) | | |

**Table S9.** Selected bond angles [°] for [W(CN$^t$Bu)$_4$(6-MePyS)(*S*–6-MePyS)] (**6c**)

| | | | |
|---|---|---|---|
| **C1-W1-S1** | 169.07(6) | **N11-W1-S1** | 63.02(5) |
| **C1-W1-N11** | 107.60(8) | **N11-W1-S2** | 74.98(5) |
| **C1-W1-S2** | 89.60(6) | **S2-W1-S1** | 82.595(18) |

| | | | |
|---|---|---|---|
| **C1-W1-C2** | 77.46(9) | **C4-W1-C3** | 72.77(9) |
| **C1-W1-C3** | 113.71(9) | **C12-S1-W1** | 81.00(8) |
| **C2-W1-S1** | 109.68(7) | **C12-N11-W1** | 104.68(14) |
| **C2-W1-N11** | 161.66(8) | **N11-C12-S1** | 111.30(17) |
| **C2-W1-S2** | 87.60(7) | **C22-S2-W1** | 118.83(8 |
| **C3-W1-S1** | 76.80(7) | **N21-C22-S2** | 113.30(16) |
| **C3-W1-N11** | 118.69(8) | **N1-C1-W1** | 177.9(2) |
| **C3-W1-S2** | 144.46(7) | **C1-N1-C31** | 165.1(2) |
| **C3-W1-C2** | 72.86(9) | **N2-C2-W1** | 179.0(2) |
| **C4-W1-S1** | 106.86(6) | **C2-N2-C35** | 168.5(2) |
| **C4-W1-N11** | 76.91(8) | **N3-C3-W1** | 177.2(2) |
| **C4-W1-S2** | 141.79(7) | **C3-N3-C39** | 176.2(3) |
| **C4-W1-C1** | 74.83(9) | **N4-C4-W1** | 175.5(2) |
| **C4-W1-C2** | 121.27(9) | **C4-N4-C43** | 155.2(2) |

**Table S10.** Selected torsion angles [°] for [W(CN$^t$Bu)$_4$(6-MePyS)(*S*–6-MePyS)] (**6c**)

| | | | |
|---|---|---|---|
| **W1-S1-C12-N11** | 0.04(14) | **W1-S2-C22-N21** | 162.14(13) |
| **W1-S1-C12-C13** | 179.1(2) | **W1-S2-C22-C23** | -19.6(2) |
| **W1-N11-C12-S1** | -0.04(17) | **C1-N1-C31-C32** | -10.5(10) |
| **W1-N11-C12-C13** | -179.19(18) | **C2-N2-C35-C36** | -139.9(12) |
| **W1-N11-C16-C15** | 177.97(16) | **C4-N4-C43-C44** | 121.4(6) |
| **W1-N11-C16-C17** | -0.8(3) | | |

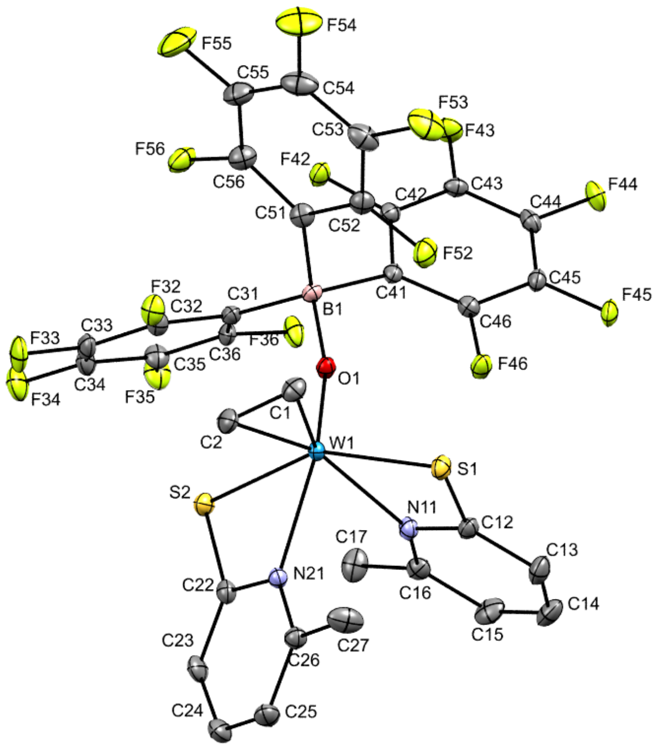


**Figure S28.** Molecular structure of [W{O(B($C_6F_5$)$_3$)}($C_2H_2$)(6-MePyS)$_2$] (**7**) showing the atomic numbering scheme. The ellipsoids are drawn at the 50% probability level. The H atoms were omitted for clarity.

**Table S11.** Selected bond lengths [Å] for [W{O(B($C_6F_5$)$_3$)}($C_2H_2$)(6-MePyS)$_2$] (**7**)

| | | | |
|---|---|---|---|
| **W1-C1** | 2.053(4) | **W1-S1** | 2.3711(9) |
| **W1-C2** | 2.065(4) | **W1-N11** | 2.306(3) |
| **W1-O1** | 1.771(3) | **W1-S2** | 2.5160(9) |
| **O1-B1** | 1.518(5) | **W1-N21** | 2.256(3) |
| **C1-C2** | 1.278(5) | | |

**Table S12.** Selected bond angles [°] for [W{O(B($C_6F_5$)$_3$)}($C_2H_2$)(6-MePyS)$_2$] (**7**)

| | | | |
|---|---|---|---|
| **B1-O1-W1** | 165.8(2) | **O1-W1-S2** | 89.32(8) |
| **C1-W1-C2** | 36.17(15) | **O1-W1-N21** | 151.92(12) |
| **C1-W1-S1** | 79.37(11) | **S1-W1-S2** | 151.35(3) |
| **C1-W1-N11** | 145.69(14) | **N11-W1-S1** | 66.40(8) |
| **C1-W1-S2** | 121.30(11) | **N11-W1-S2** | 90.41(8) |
| **C1-W1-N21** | 103.78(14) | **N21-W1-S1** | 92.50(8) |
| **C2-W1-S1** | 109.92(12) | **N21-W1-N11** | 76.60(11) |

| | | | |
|---|---|---|---|
| **C2-W1-N11** | 159.35(14) | **N21-W1-S2** | 64.74(8) |
| **C2-W1-S2** | 85.66(12) | **C2-C1-W1** | 72.4(3) |
| **C2-W1-N21** | 83.40(13) | **C1-C2-W1** | 71.4(3) |
| **O1-W1-C1** | 98.77(14) | **B1-O1-W1** | 165.8(2) |
| **O1-W1-C2** | 106.24(14) | **O1-B1-C31** | 103.7(3) |
| **O1-W1-S1** | 108.06(8) | **O1-B1-C41** | 109.9(3) |
| **O1-W1-N11** | 93.96(12) | **O1-B1-C51** | 107.3(3) |

# 8 References

(1) Ehweiner, M. A.; Peschel, L. M.; Stix, N.; Ćorović, M. Z.; Belaj, F.; Mösch-Zanetti, N. C. Bioinspired Nucleophilic Attack on a Tungsten-Bound Acetylene: Formation of Cationic Carbyne and Alkenyl Complexes. *Inorg. Chem.* **2021**, *60* (12), 8414–8418. DOI: 10.1021/acs.inorgchem.1c00643

(2) Ehweiner, M. A.; Ćorović, M. Z.; Belaj, F.; Mösch-Zanetti, N. C. Synthesis and Reactivity of Molybdenum and Tungsten Alkyne Complexes Containing 6-Methylpyridine-2-thiolate Ligands. *Helv. Chim. Acta* **2021**, *104* (11), e2100137. DOI: 10.1002/hlca.202100137

(3) Lancaster, S. *Alkylation of boron trifluoride with pentafluorophenyl Grignard reagent; Tris(pentafluorophenyl)boron; borane*. http://cssp.chemspider.com/Article.aspx?id=215, **2010**

(4) Dub, P. A.; Filippov, O. A.; Belkova, N. V.; Rodriguez-Zubiri, M.; Poli, R. Experimental (IR, Raman) and computational analysis of a series of $PtBr_2$ derivatives: vibrational coupling in the coordinated ethylene and Pt-Br modes. *J. Phys. Chem. A* **2009**, *113* (22), 6348–6355. DOI: 10.1021/jp902394j

(5) Kanishchev, O. S.; Dolbier, W. R. Synthesis and characterization of 2-pyridylsulfur pentafluorides. *Angew. Chem. Int. Ed.* **2015**, *54* (1), 280–284. DOI: 10.1002/anie.201409990

(6) Agapie, T.; Schofer, S. J.; Labinger, J. A.; Bercaw, J. E. Mechanistic studies of the ethylene trimerization reaction with chromium-diphosphine catalysts: experimental evidence for a mechanism involving metallacyclic intermediates. *J. Am. Chem. Soc.* **2004**, *126* (5), 1304–1305. DOI: 10.1021/ja038968t

(7) Tadros, S. M.; Mansour, M.; Naik, D. V.; Moehring, G. A. Line Shape Analysis of Dynamic NMR Spectra for Characterizing Coordination Sphere Rearrangements at a Chiral Rhenium Polyhydride Complex. *J. Vis. Exp.* **2022** (185), e64160. DOI: 10.3791/64160

(8) Bruker. *APEX2*; Bruker AXS Inc., 2012

(9) Bruker. *SADABS*; Bruker AXS Inc., 2016

(10) Sheldrick, G. M. SHELXT - integrated space-group and crystal-structure determination. *Acta Cryst.* **2015**, *A71*, 3–8. DOI: 10.1107/S2053273314026370

(11) Sheldrick, G. M. A short history of SHELX. *Acta Cryst.* **2008**, *A64*, 112–122. DOI: 10.1107/S0108767307043930

(12) Sheldrick, G. M. Crystal structure refinement with SHELXL. *Acta Cryst.* **2015**, *71*, 3–8. DOI: 10.1107/S2053229614024218

(13) Dolomanov, O. V.; Bourhis, L. J.; Gildea, R. J.; Howard, J. A. K.; Puschmann, H. OLEX2: a complete structure solution, refinement and analysis program. *J. Appl. Crystallogr.* **2009**, *42* (2), 339–341. DOI: 10.1107/S0021889808042726
